# Combined Effects of Microplastics and Mercury on Growth, Hematology, Tissue Morphology, GH/IGF Axis, and Antioxidant–Immune Responses in Nile Tilapia

**DOI:** 10.1155/jt/1847474

**Published:** 2026-06-19

**Authors:** Md Ruhul Amin, Moshfiq Momtasir Neloy, Tasmia Islam Kanta, Md. Mahiuddin Zahangir, Saleha Khan, Md Shahjahan

**Affiliations:** ^1^ Laboratory of Fish Ecophysiology, Department of Fisheries Management, Bangladesh Agricultural University, Mymensingh, 2202, Bangladesh, bau.edu.bd; ^2^ Department of Fisheries Technology, Bangladesh Agricultural University, Mymensingh, 2202, Bangladesh, bau.edu.bd; ^3^ Department of Fish Biology and Biotechnology, Faculty of Fisheries, Chattogram Veterinary and Animal Sciences University, Chattogram, 4225, Bangladesh, cvasu.ac.bd

**Keywords:** bioaccumulation, blood biomarkers, environmental risk, mercury, microplastics

## Abstract

Microplastics (MPs) and heavy metal mercury (Hg) have drawn global surveillance as major contaminants due to their toxic effects on aquatic organisms. The individual effects of both contaminants have been extensively characterized; however, their coexposure effects remain insufficiently explored. As aquatic organisms are increasingly exposed to multiple pollutants simultaneously in natural environments, this investigation explored the combined effects of polyamide MP (PA‐MP) and Hg on Nile tilapia (*Oreochromis niloticus*) fingerlings, focusing on survival, growth, hematological balance, tissue structure, GH/IGF axis regulation, and immune–antioxidant responses. Over a 42‐day trial, 240 Nile tilapia fingerlings were allocated into four triplicated treatments: control (no PA‐MP or Hg), PA‐MP (10 mg/L), Hg (0.03 mg/L), and PA‐MP + Hg (10 mg/L + 0.03 mg/L), with 20 fingerlings per tank. The coexposure group showed increased MP accumulation and mortality, suppressed growth indicators, and substantial shifts in blood physiology, including elevated glucose (126.83 ± 2.40 mg/dL) and lowered hemoglobin (9.45 ± 0.85 g/dL), along with higher cellular and nuclear abnormalities. The histoarchitectural assessment identified severe structural deformities in the gills, intestine, liver, and kidney of coexposed fish compared to control and individual contaminants. At the transcriptional level, the expression of growth hormone‐secreting gene (*gh*) in the pituitary and insulin‐like growth factors (*igf-1* and *igf-2*) in the liver showed a significant downregulation under coexposure treatment. Moreover, coexposure to PA‐MP and Hg induced hepatic oxidative damage by affecting antioxidant defense, as evidenced by altered activity of superoxide dismutase (*sod*) and catalase (*cat*), and simultaneously modulated immune responses by significantly upregulating interferon‐γ (*ifn-γ*) and tumor necrosis factor‐α (*tnf-α*) while downregulating interleukin‐1β (*il-1β*), indicating an oxidative–inflammatory response. These outcomes collectively underscore that MP and Hg coexposure aggravates both systemic and molecular impairments in Nile tilapia, resulting in weakened molecular responses, impaired physiological functions, and decreased survivability.

## 1. Introduction

Microplastics (MPs; tiny particles < 5 mm), as an emerging environmental contaminant, are increasingly prevalent in the marine, freshwater, and terrestrial environments due to the overuse of plastic and inadequate disposal practices. Domestic wastewater discharges allow primary MPs, such as those found in cosmetics and personal care products, including toothpaste, soap, shampoo, and cleanser, to enter aquatic habitats [1]. Secondary MPs, generated by the fragmentation of larger plastics into smaller particles within aquatic systems through abrasive forces, solar radiation, and biodegradation, further exacerbate MP contamination due to their persistence, mobility, and continuous accumulation over time [2]. Fine particulate MPs are ingested by diverse organisms through several modes, such as direct consumption, surface adherence, and trophic transfer. Consequently, MPs in aquatic environments have incited widespread concern due to their detrimental effects on organisms [3], as exposure can result in impaired hematology, tissue damage, endocrine disruption, oxidative stress, and immune dysfunction, ultimately leading to stunted growth and mortality [4–6].

Heavy metal (HM) pollution is another significant environmental threat because it is persistent and ubiquitous in aquatic environments. They are introduced into water bodies through anthropogenic activities, including mining operations, metal processing, and industrial effluents [7], as well as from natural sources such as erosion, volcanic emissions, and wildfires [8]. Their pervasive presence in the aquatic environment presents substantial ecological risks due to their nonbiodegradable and bioaccumulative nature and toxicity in aquatic biota [9]. Among these, mercury (Hg) is one of the most critical contaminants due to its long‐range transport, high toxicity even at low concentrations, and its extended biological persistence within organisms [10]. In environmental matrices, Hg occurs in three major forms, namely elemental Hg (Hg^0^), inorganic Hg (Hg^+^ or Hg^2+^), and organic Hg (predominantly methylmercury [MeHg]), all of which pose significant toxicological risks to aquatic life [11]. Hg is a biologically nonessential HM for fish, with a pronounced affinity for organs (e.g., gills and liver), where it accumulates via respiration, feeding, and absorption through the skin and mucus membranes [12]. This accumulation causes disturbances in biological processes such as hematological changes, organ damage, oxidative stress, genotoxicity, immune dysregulation, cardiotoxic effects, and overall impairment of fish well‐being [10, 13].

In aquatic environments, MPs do not exist in isolation but act as vectors for potentially toxic elements (PTEs), forming combined contaminants that intensify ecotoxic responses in aquatic organisms [14]. Due to their nonpolar surface, high stability, and sorption affinity, MPs can accumulate chemical contaminants, including pharmaceuticals, HMs, pesticides, and persistent organic pollutants [15]. The combination of their minute size and extensive surface area enables them to transport toxic chemicals to aquatic organisms, facilitating pollutant bioaccumulation and intensifying toxicity through trophic transfer [16]. Accordingly, growing attention is being directed toward the interactive effects of MPs and chemical toxicants on animal physiology, particularly their impact on ingestion rates, tissue accumulation, and multiple physiological and biochemical responses [17, 18].

Exposure to toxicants, whether HMs or MPs, can elicit stress responses at distinct biological tiers, including molecular, genetic, cellular, and tissue modifications, as well as changes in somatic growth, physiological functions, and survivability. In toxicological studies, hematobiochemical indices such as glucose and hemoglobin concentrations are stress indicators of fish health, as blood transports environmental toxicants throughout the body [4, 19, 20]. Furthermore, nuclear and cellular irregularities in erythrocytes are reliable indicators of genotoxic effects exerted by toxicant exposure [21]. For example, exposure to subacute concentrations of Cr and polyamide MP (PA‐MP), individually, has been reported to exert stress and genotoxicity in fish by altering serum glucose, hemoglobin, and erythrocyte morphology [22]. The expression profiles of antioxidant–immune genes are prominent and sensitive molecular markers that can be used to evaluate oxidative stress and immunotoxicity in response to environmental pollutants, as well as monitor piscine health [23, 24]. Furthermore, histopathological alterations in internal tissues (e.g., gills, kidney, liver, and intestine) represent the severity of pollutant‐induced cytotoxicity. Reports indicate that the accumulation of Hg or MP exerts histoarchitectural changes in major organs (e.g., gills, liver, kidney, and intestine) of fish [2, 13]. Therefore, the toxicological impact of their combined exposure remains a major concern in aquatic ecosystems.

Nile tilapia (*Oreochromis niloticus*) ranks as the third most popular farmed species worldwide due to its rapid growth, high adaptability, and economic significance in aquaculture. Beyond its economic value, this species has been accepted as a reliable bioindicator in ecotoxicological assessments [25]. PA, commonly known as nylon 6, is widely utilized in aquaculture operations for the production of fishing gear and equipment [26]. Discarded or lost gear undergoes weathering over time, contributing to the accumulation of PA‐MP in aquatic environments, which has become a pressing environmental concern. Simultaneously, Hg is a pervasive HM with high toxicity even at low concentrations and a strong tendency to bioaccumulate in fish tissues [13]. Ecotoxicological investigations have unveiled the detrimental effects of PA‐MP [27] or Hg exposure [28] on Nile tilapia fingerlings. Although the impacts of single contaminants have been widely studied, the combined effects of PA‐MP and Hg exposure are still inadequately elucidated, particularly regarding physiological performance, biochemical and hormonal responses, and bioaccumulation patterns. Addressing this knowledge gap is critical for understanding potential risks in aquaculture systems and for devising mitigation strategies that safeguard fish health and food safety. Therefore, this study aimed to systematically evaluate the concurrent effects of PA‐MP and Hg on growth performance, feeding efficiency, survival, hematology, tissue morphology, antioxidant–immune gene expression, GH/IGF axis regulation, and accumulation patterns in Nile tilapia.

## 2. Materials and Methodology

### 2.1. Source of MPs and Hg

The virgin PA‐MPs were synthesized from unused toothbrush bristles according to the procedure outlined by Xie et al. [29]. An electric trimmer (Philips Trimmer; Model BT1230, China) fitted with high‐precision stainless‐steel blades was used to refine the bristles down to the smallest achievable dimensions. Particle samples were sieved through a stainless‐steel strainer with four mesh sieves (2 mm, 1 mm, 500 μm, and 125 μm). MP size was reexamined by analyzing particle dimensions using a calibrated microscope (AmScope MU1003, China) with 4× magnification. Thereafter, the captured images were examined to classify particle dimensions and assign the MP to the appropriate size categories. Lastly, PA‐MP particles were quantified by weighing, and the groups were mixed accordingly in proportional ratios before initiating the experimental trial. The processes of cutting, separation, and measurement were carried out within a ductless fume cupboard to prevent cross‐contamination. MP particles obtained from unused toothbrush bristles were confirmed as PA6 and PA66 using ATR‐FTIR [27]. In this trial, the MP size range (125 μm–2 mm) was designated based on previous investigations [4, 30], which indicate that MPs (125 μm–4 mm) are ubiquitously found in aquatic ecosystems and tend to accumulate in aquatic organisms, including fish.

EMSURE, analytical‐grade mercuric chloride (HgCl_2_) from Supelco (Darmstadt, Germany; Cat. No. 1.04419.0050), was obtained from a licensed chemical supplier in Dhaka, Bangladesh, and used as the source of Hg for the experiment. In this study, a concentrated Hg stock solution was formulated by dissolving 1.35 g of HgCl_2_ in 1 L of distilled water to obtain a final concentration of approximately 1 mg/mL. A 0.03 mg/L Hg working solution was prepared from the stock solution by diluting 0.03 mL of stock to 1 L with distilled water.

### 2.2. Experimental Animals and Acclimatization

Healthy, pathogen‐free, and responsive monosex Nile tilapia (*O. niloticus*) fingerlings (*n* = 240) of homogeneous size and weight (mean weight: 5.05 ± 0.63 g; mean length: 6.34 ± 0.54 cm) were procured from Sarnolota Hatchery, Mymensingh. The fingerlings were carefully handled and transported to the Laboratory of Fish Ecophysiology under regulated conditions to attenuate handling stress. Before the onset of the exposure period, the fingerlings underwent acclimatization in a large 1500‐L rectangular cemented tank at an ambient temperature of 26 ± 0.08°C with a natural photoperiod (LD—14:10 h) and regular aeration for 15 days. During the habituation period, the fingerlings were fed to satiation twice per day (at 9:00 a.m. and 5:00 p.m.) with a commercial diet (containing 32% protein) specifically formulated for tilapia rearing at a feeding rate of 5% of their body weight.

### 2.3. Exposure Protocols

This investigation was carried out in accordance with OECD Test No. 215: Fish, Juvenile Growth Test [31], which prescribes a 28‐day rearing period to evaluate sublethal effects on growth and provide adequate exposure for detecting potential toxicological impacts in juvenile fish. Postacclimatization, the Nile tilapia (*O. niloticus*) fingerlings (*n* = 20 fingerlings/tank) were randomly assigned to four triplicated experimental glass aquaria (*n* = 12 tanks; dimensions: 75 cm length × 45 cm width × 45 cm height) labeled as control (without MP or Hg), MP (10 mg/L), Hg (0.03 mg/L), and MP + Hg (10 mg/L + 0.03 mg/L). Each experimental unit was filled with 100 L of groundwater and sustained for a period of 42 days. In line with OECD guidelines and considering the market importance of Nile tilapia, the rearing period in the current study was extended by an additional 2 weeks to facilitate improved growth measurement. During toxicant exposure, the fingerlings received a standard commercial feed (0.8‐mm floating pellets; containing 24% carbohydrate, 15% ash, 12% moisture, 8% lipid, 3% fiber, 3% calcium, and phosphorus) at 5% of their body weight, administered twice daily (at 9:00 a.m. and 5:00 p.m.). Fingerlings were provided a feeding period of 1 h, after which residual feed and waste materials were promptly siphoned out. Aeration of the experimental glass aquaria was maintained using an air pump to secure sufficient dissolved oxygen (DO) levels and facilitate even distribution of test ingredients through water agitation. To ensure the precision of PA‐MP and Hg concentrations, the test solutions were replaced at 2‐day intervals, and the tanks were cleaned with precision. Additionally, water temperature (28−29°C), pH (8.1−8.3), and total ammonia nitrogen (TAN) concentrations (0.25−0.50 ppm) were maintained at optimum levels throughout the exposure study. At the end of the 42‐day trial, fish were sampled to determine MP accumulation patterns, growth performance, feeding efficiency, and alterations in selected physiological parameters. The entire experimental operations, including toxicant exposure and animal maintenance, were approved by the Animal and Ethical Committee at Bangladesh Agricultural University, Mymensingh 2202, Bangladesh (No. BAU‐FoF/2022/002).

The present study designated an Hg concentration of 0.03 mg/L, equivalent to 1/10th of the 96‐h LC_50_ value documented by Jasim et al. [28] for Nile tilapia. This framework is routinely adopted to evaluate the initial physiological impairments due to toxicant challenge [32]. In addition, a 10 mg/L PA‐MP concentration was introduced into the experimental rearing aquaria, as this concentration has previously been documented to cause alterations in blood profiles and tissue morphology of fish [4, 27]. The PA‐MP concentration of 10 mg/L was used in this study and determined from the concentrations reported in severely polluted environments [33, 34]. This concentration allows precise detection of physiological and transcriptional responses under regulated exposure conditions and represents environmentally realistic long‐term exposure conditions.

### 2.4. MP Extraction and Analysis

From each test glass aquarium, six (*n* = 6) fish were randomly sampled and euthanized using clove oil at a concentration of 5 mg/L for 10 min. The vital organs, including gills and gut, were carefully excised through a ventral incision, collected, and weighed (g). The gill and gut organic matter were digested separately in beakers containing analytical‐grade KOH (10% w/v), and aluminum foil was used to cover each beaker to prevent contamination. To facilitate complete decomposition of organic matter, the beakers were maintained on heated magnetic stirrers at 50°C for 72 h. Following digestion, each beaker received a saturated 30% NaCl solution (1 mL per 1 mL of digestion solution) to achieve density separation. The final solution was then decanted through a cellulose nitrate filter (mesh size: 0.45 μm, Sartorius) using a vacuum filtration unit (Duran, Germany) connected to a vacuum pump (Rocker 300, Taiwan). Finally, the filter papers were preserved in Petri dishes and covered with aluminum foil for later microscopic observation. The entire procedure was carried out in a fume hood to minimize the risk of cross‐contamination. MP particles retained on the filter papers were quantified using a microscope (Micros, MCX100, Austria; AmScope, MU1003, China).

### 2.5. Estimation of Growth Indices, Feeding Efficiency, and Survival

After a 42‐day exposure period, the fingerlings underwent a 24‐h feed‐deprivation period; afterward, body weights were assessed with an electronic balance and noted. Growth outcomes and feed utilization patterns were assessed using the following equations:

(1) Weight gaing=Final body weight g−initial body weight g,

(2) Specific growth rate%day=ln  final weightg−ln  initial weightgExperimental duration days×100,

(3) Feed conversion ratioFCR=Feed given dry weightBody weight gain wet weight,

(4) Survival rate %=Total numer of fish survived at the end of the exposureInitial number of fish stocked×100.

### 2.6. Estimation of Serum Biochemical Parameters and Erythrocyte Morphology

To assess the shifts in serum biochemical indices, including glucose and hemoglobin, eight fish (*n* = 8) were selected randomly and euthanized from each experimental glass aquarium. The fingerlings were immediately anesthetized with clove oil (5 mg/L), and blood specimens were sampled from the caudal peduncle with the aid of a heparinized syringe. Glucose (mg/dL) and hemoglobin (g/dL) concentrations were measured immediately using glucose and hemoglobin test strips with a digital EasyMate GHb integrated monitoring device (Model: ET‐232, Bioptik Technology Inc., Taiwan 3505‐7).

In addition, blood samples (3 μL) were obtained from the caudal region of each fish (*n* = 10), immediately smeared on sterilized glass slides, and air‐dried for 15 min for erythrocytic anomaly examination. Following air drying, smears were immersed in undiluted ethanol (99.99%) for 10 min, stained with 5% Giemsa’s solution for another 10 min, and subsequently rinsed with purified water. Following staining, blood smears were observed at 40x magnification with a Micros MCX100 microscope (Austria). Six blood smears (*n* = 6) were analyzed per fish, with approximately 500 erythrocytes evaluated on each slide. Micrographs were obtained with a digital microscopic camera (AmScope MU1003, China) to document the erythrocytic anomalies (cellular and nuclear) as described by Hasan et al. [27].

### 2.7. Tissue Preservation, Processing, and Morphological Observation

For histometric assessment of vital organs, six fish (*n* = 6) were anesthetized with clove oil (5 mg/L) and euthanized, followed by a ventral incision at the end of the exposure study. The gills, liver, kidney, and intestine were carefully excised under aseptic conditions to minimize the risk of cross‐contamination. Before tissue processing, the specimens were stabilized in a fixative agent (Bouin’s solution) for 24 h and then transferred to labeled vials containing 70% ethanol and kept in a refrigerator (4°C). Afterward, the specimens underwent a graded ethanol series (80%, 90%, 95%, and 100%), and histological slides were prepared in accordance with standard procedures (paraffin embedding, microtome sectioning, staining, and DPX mounting) as reported by Feldman et al. [35]. The prepared histological slides (10 slides/treatment) were examined using a microscope (Micros, MCX100, Austria), and organ‐specific tissue abnormalities were imaged with a camera (AmScope MA1000, China) to facilitate visual and semiquantitative analysis. The abnormalities were graded as “−“ = 0% (no abnormalities), “+” = < 10% (mild abnormalities), “++” = 10%–50% (moderate alterations), and “+++” = > 50% (severe abnormalities) according to Shahriar et al. [4].

### 2.8. RNA Isolation, cDNA Synthesis, and Real‐Time Polymerase Chain Reaction (PCR) (RT‐PCR) Assay

For transcriptomic profiling, the pituitary glands and liver were separated from fingerlings at the termination of the exposure, promptly placed in Eppendorf tubes containing 1000 μL of RNAlater (Sigma‐Aldrich, Germany) to stabilize RNA, and stored at 4°C for 24 h before transfer to −80°C. Each sample was lysed with 1 mL of TRIzol reagent (Invitrogen, Thermo Fisher, USA) for RNA extraction and homogenized using an electric mortar (Stuart, Germany). The lysate was centrifuged at 4°C for 5 min, followed by chloroform addition to induce phase separation; following precipitation with isopropanol, RNA was washed in 75% ethanol, air‐dried, resuspended in DEPC‐treated water, and preserved at −80°C for further examination. RNA purity and concentration were measured using a NanoDrop photometer (NP80, Implen, Germany) at a 260/280 absorbance ratio. First‐strand cDNA was synthesized from ∼1000 ng of total RNA using a cDNA synthesis kit (AddScript cDNA Synthesis Kit, Korea), following company standards. Transcriptional inversion was carried out using a thermal exposure protocol that included 25°C for 10 min (primer annealing), 50°C for 60 min (reverse transcription), and 80°C for 5 min (enzyme inactivation). The primers designed to quantify the growth and antioxidant–immune‐related gene expression patterns are outlined in Table 1.

**TABLE 1:** List of primers used in the real‐time PCR.

Gene name	Primer sequence (5′–3′)	Reference
*Growth-related genes*		
*gh*	F—CTGCTGATCAGGGCCAATC R—TCGACATTTAGCTACCGTCAGG	[36]

*igf-1*	F—GTTTGTCTGTGGAGAGCGAGG R—GAAGCAGCACTCGTCCACG	[36]

*igf-2*	F—GCTTTTATTTCAGTAGGCCAACCA R—CACAGCTACAGAAAAGACACTCCTCTA	[36]

*Antioxidant-related genes*		
*sod*	F—GACGTGACAACACAGGTTGC R—TACAGCCACCGTAACAGCAG	[37]

*cat*	F—TCAGCACAGAAGACACAGACA R—GACCATTCCTCCACTCCAGAT	[37]

*Immune-related genes*		
*il-1β*	F—CAAGGATGACGACAAGCCAACC R—AGCGGACAGACATGAGAGTGC	[37]

*ifn-γ*	F—TGACCACATCGTTCAGAGCA R—GGCGACCTTTAGCCTTTGT	[37]

*tnf-α*	F—GGAAGCAGCTCCACTCTGATGA R—CACAGCGTGTCTCCTTCGTTCA	[37]

*Reference gene*		
*β-actin*	F—CGAGCTGTCTTCCCATCCA R—TCACCAACGTAGCTGCTTTCTG	[38]

*Note:* F: forward; R: reverse.

Afterward, PCR was performed in a total of 10 μL reaction mixture containing 1 μL of cDNA template, 0.4 μL of each primer, 5 μL of TB Green Premix Ex *Taq* II, and 3.2 μL of RNase‐free water. RT‐PCR quantification was performed using the QuantStudio 5 system (Applied Biosystems, USA) under the following amplification steps, which included initial denaturation at 95°C for 30 s (one cycle), followed by 40 cycles of denaturation at 95°C for 5 s, primer annealing at 60°C for 30 s, and extension at 72°C for 1 min. The accuracy of reverse transcription was verified through melting curve analysis. Additionally, the 2^−ΔΔCT^ method was employed to quantify gene expression levels using *β-actin* as the reference gene; each qPCR assay was conducted in triplicate to ensure reliability.

### 2.9. Statistical Analysis and Visualization

The results are presented as mean ± standard deviation (SD) and standard error of the mean (SEM). The Shapiro–Wilk test was applied to evaluate normality, whereas Levene’s test was used to assess homogeneity of variance. Statistical significance was set at *p* < 0.05; group differences were evaluated by one‐way ANOVA with Tukey’s *HSD* post hoc analysis using SPSS version 26.0 (SPSS Inc., Chicago, IL). To determine the overall efficacy of the treatments, principal component analysis (PCA) was conducted based on the mean values of the assessed parameters. Data visualization was performed using Python 3.11.3 (https://www.python.org).

## 3. Results

### 3.1. MP Ingestion

**FIGURE 1:**
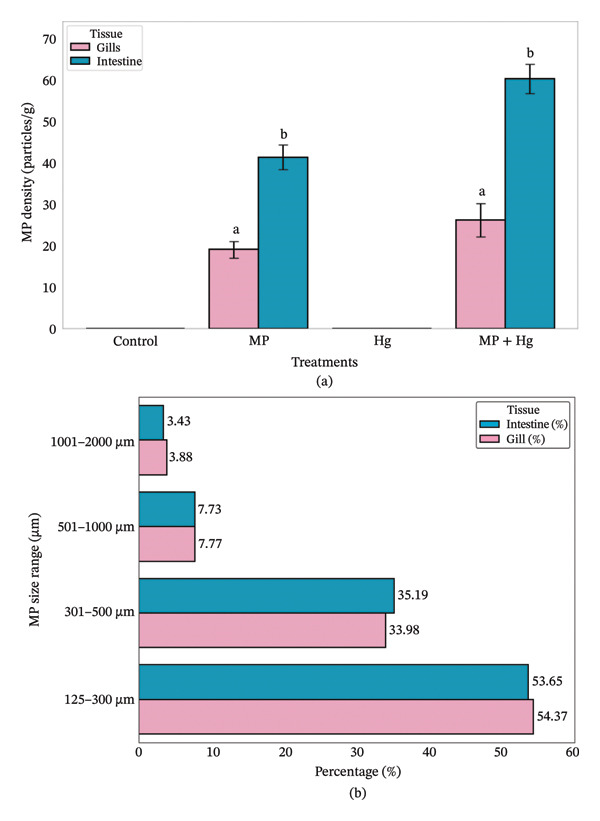
(a) The abundance and (b) size distribution percentages of MP isolated from the GIT and gills of Nile tilapia; data are expressed as mean ± standard deviation (SD); superscripts (a, ab, bc, b, and c) indicate significant variation (*p* < 0.05) among treatments; MP: microplastic.

MP particles were detected in the gills and gastrointestinal tract (GIT) of fish subjected to MP and MP + Hg test conditions; in contrast, no MPs were observed in fish reared under Hg and control conditions. Fish reared under MP + Hg condition showed a significantly elevated accumulation of MPs in GIT (59.67 ± 8.50 g^−1^) and gills (25.83 ± 3.06 g^−1^) compared to those reared under MP condition (41.17 ± 3.31 g^−1^ in GIT and 18.67 ± 1.97 in gills; *p* < 0.05; Figure 1a). The most prevalent MP size range identified in both GIT (53.65%) and gills (54.37%) was 125–300 μm, followed by 301–500 μm (35.19% and 33.98%, respectively; Figure 1b), but larger sizes (501–2000 μm) were detected in much lower proportions.

### 3.2. Growth Indices, Feeding Efficiency, and Survival

Table 2 demonstrates the effects of MP and Hg (alone or combined) on the growth and feed efficiency performance of Nile tilapia (*O. niloticus*) in terms of final body weight (FBW), weight gain (WG), specific growth rate (SGR), feed conversion ratio (FCR), and net survival under distinct test conditions. The highest values of FBW, WG, and SGR were measured in the untreated condition, but the lowest values were observed under combined exposure to MP and Hg. The mean FBW was highest in the control group, whereas significantly lower values (*p* < 0.05) were recorded in MP, Hg, and MP + Hg treatments, respectively. A uniform trend was observed for WG and SGR with the highest values in the control group and lower values in MP, Hg, and MP + Hg treatments, which indicates severe growth suppression due to the toxic effects of these stressors. Furthermore, the FCR was most favorable in the control group and the exposed groups showed impaired feed utilization with a gradual increase in MP, Hg, and the highest values in MP + Hg, indicating diminished dietary efficiency. Mean survival was lowest in the MP + Hg treatment (*p* < 0.05) followed by the Hg treatment; no mortality occurred in the MP and control conditions.

**TABLE 2:** Growth indices, feed conversion, and survivability percentages (%) of Nile tilapia exposed to MP and Hg treatments for 42 days.

Parameters	Treatments			
	Control	MP	Hg	MP + Hg
IBW (g)	5.06 ± 0.79^a^	5.05 ± 0.63^a^	5.02 ± 0.75^a^	5.05 ± 0.66^a^
FBW (g)	23.38 ± 3.70^b^	19.42 ± 2.93^a^	17.68 ± 3.85^a^	16.82 ± 3.62^a^
WG (g)	18.32 ± 3.70^b^	14.37 ± 2.93^a^	12.66 ± 3.85^a^	11.77 ± 3.62^a^
SGR (%/day)	3.62 ± 0.37^b^	3.18 ± 0.40^ab^	2.95 ± 0.51^a^	2.80 ± 0.58^a^
FCR	1.25 ± 0.25^a^	1.65 ± 0.49^a^	2.01 ± 0.61^ab^	2.54 ± 1.22^b^
Survival (%)	100^c^	100^c^	91.23 ± 3.04^b^	78.95 ± 3.04^a^

*Note:* MP: microplastic; Hg: mercury (Hg); data are expressed as mean ± standard deviation (SD); superscripts (a, ab, b, and c) indicate significant variation (*p* < 0.05) among treatments.

Abbreviations: FBW, final body weight; FCR, feed conversion ratio; IBW, initial body weight; SGR, specific growth ratio; WG, weight gain.

### 3.3. Serum Biochemical Indices

**FIGURE 2:**
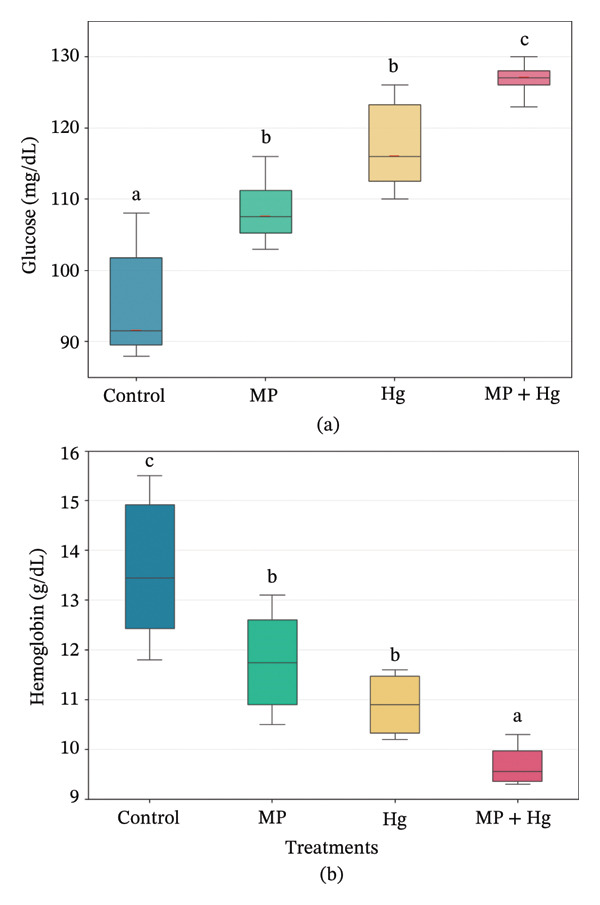
(a) Serum glucose and (b) hemoglobin concentrations of Nile tilapia exposed to MP and Hg treatments for 42 days; data are expressed as mean ± standard deviation (SD); superscripts (a, ab, bc, b, and c) indicate significant variation (*p* < 0.05) among treatments; MP: microplastic; Hg: mercury.

Figures 2a and b depict the variations in two serum biochemical indices, glucose (mg/dL) and hemoglobin (g/dL), in the blood of Nile tilapia (*O. niloticus*) subjected to different test conditions of MP and Hg. The mean glucose concentration was lowest in the untreated condition, varying significantly from MP (*p* < 0.05). Serum glucose levels were significantly elevated (*p* < 0.05) in Hg and MP + Hg test conditions compared with the control; although MP and Hg did not vary significantly (*p* > 0.05) from each other, this indicates MP exposure and Hg exposure (alone or combined) disrupt glucose regulation in fish. Besides, hemoglobin concentration significantly declined (*p* < 0.05) in the MP + Hg and Hg test conditions compared with the control, whereas the MP did not vary significantly (*p* > 0.05) from each other, and this trend indicates hematological stress under MP and Hg exposure.

### 3.4. Structural Changes in Erythrocyte Morphology

**FIGURE 3:**
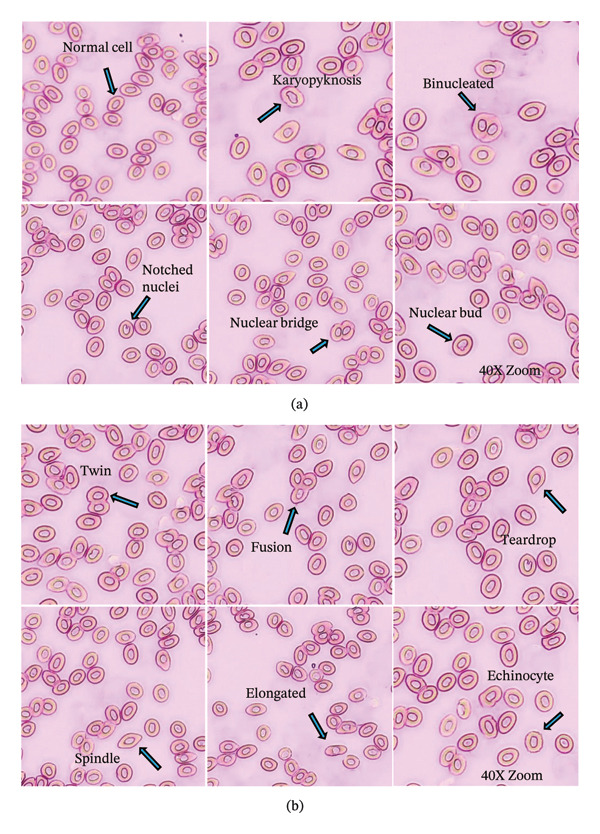
An illustration of erythrocytic abnormalities: (a) nuclear (ENA) and (b) cellular (ECA) alterations observed in Nile tilapia to MP and Hg treatments for 42 days; MP: microplastic; Hg: mercury.

Table 3 presents the documented frequencies of erythrocytic abnormalities, including erythrocytic cellular abnormality (ECA) and erythrocytic nuclear abnormality (ENA), across the various test conditions. We identified five distinct forms of nuclear abnormalities (karyopyknosis, binucleated, notched nuclei, nuclear bridge, and nuclear bud; Figure 3a) and six different types of cellular abnormalities (twin, fusion, teardrop, spindle, elongated, and echinocyte; Figure 3b) in erythrocytes as a result of MP and Hg exposure. Nuclear and cellular abnormalities occurred most frequently in the MP + Hg group, followed by the Hg treatment, and the MP treatment, which indicates that exposure to MP and Hg (alone or combined) modifies erythrocyte morphology by exerting adverse effects.

**TABLE 3:** The frequency of erythrocytic (cellular and nuclear) abnormalities in Nile tilapia exposed to MP and Hg treatments for 42 days.

Erythrocytic abnormalities	Treatments			
	Control	MP	Hg	MP + Hg
*Nuclear abnormalities*				
Karyopyknosis	0.16 ± 0.05^a^	0.38 ± 0.06^a^	0.48 ± 0.11^ab^	0.82 ± 0.13^b^
Binucleated	0.22 ± 0.04^a^	0.34 ± 0.07^a^	0.46 ± 0.08^a^	0.74 ± 0.07^b^
Notched nuclei	0.26 ± 0.05^a^	0.50 ± 0.08^ab^	0.74 ± 0.08^bc^	1.02 ± 0.08^c^
Nuclear bridge	0.18 ± 0.05^a^	0.20 ± 0.04^ab^	0.28 ± 0.06^ab^	0.42 ± 0.08^b^
Nuclear bud	0.28 ± 0.06^a^	0.48 ± 0.10^ab^	0.66 ± 0.07^bc^	0.96 ± 0.09^c^

*Cellular abnormalities*				
Twin	0.48 ± 0.06^a^	0.72 ± 0.10^ab^	1.06 ± 0.07^bc^	1.34 ± 0.13^c^
Fusion	0.52 ± 0.06^a^	0.82 ± 0.08^a^	1.16 ± 0.07^b^	1.22 ± 0.12^b^
Teardrop	0.32 ± 0.04^a^	0.66 ± 0.11^b^	0.98 ± 0.08^bc^	1.26 ± 0.10^c^
Spindle	0.38 ± 0.09^a^	0.72 ± 0.06^b^	1.18 ± 0.08^c^	1.32 ± 0.10^c^
Elongated	0.36 ± 0.04^a^	0.64 ± 0.07^a^	0.96 ± 0.11^b^	1.24 ± 0.08^b^
Echinocyte	0.42 ± 0.05^a^	0.82 ± 0.06^b^	1.08 ± 0.07^b^	1.38 ± 0.11^c^

*Note:* MP: microplastic; Hg: mercury (Hg); data are expressed as mean ± standard error of the mean (SEM); superscripts (a, ab, b, and c) indicate significant variation (*p* < 0.05) among treatments.

### 3.5. Structural Changes in Tissue Morphology

#### 3.5.1. Structural Changes in Gills

**FIGURE 4:**
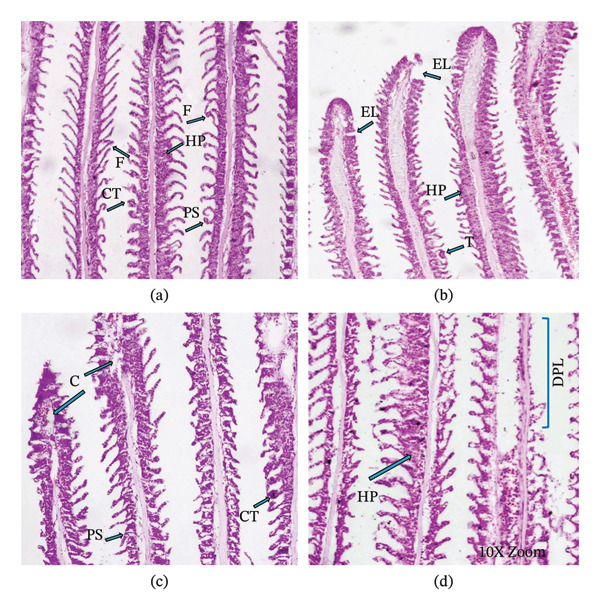
(a–d) The histoarchitectural alterations in gills of Nile tilapia exposed to MP and/or Hg: epithelial lifting (EL), lamellar fusion (F), hyperplasia (HP), telangiectasia (T), congestion in primary lamellae (C), deformed pillar system (PS), clubbed tips (CT), degeneration in primary lamellae (DPL); MP: microplastic; Hg: mercury.

Table 4 summarizes the semiquantitative profiling of histopathological changes in the organs, including gills, intestine, liver, and kidney, after 42 days of MP and Hg exposure (alone or combined). Under light microscopy, the gill tissue of Nile tilapia (*O. niloticus*) in the control group showed intact structures, except for mild (< 10%) hyperplasia. In *O*. *niloticus* treated solely with Hg, gill tissue damage was observed, primarily characterized by moderate (10−50%) epithelial lifting, lamellar fusion, hyperplasia, telangiectasia, congestion in primary lamellae, deformed pillar system, clubbed tips, and degeneration in primary lamellae (Figure 4). However, in the MP + Hg exposure and MP groups, more severe (> 50%) histopathological alterations were found, including lamellar fusion, hyperplasia, deformed pillar system, and degeneration in primary lamellae.

**TABLE 4:** Semiquantitative data of histoarchitectural changes in the gills, intestine, liver, and kidneys due to exposure to MP and/or Hg for 42 days.

Tissue abnormalities	Treatments			
	Control	MP	Hg	MP + Hg
*Gill abnormalities*				
Epithelial lifting (EL)	−	++	+	++
Lamellar fusion (F)	−	+++	+	+++
Hyperplasia (HP)	+	+++	++	+++
Telangiectasia (T)	−	++	+	+++
Congestion in primary lamellae (C)	−	+	−	++
Deformed pillar system (PS)	−	++	+	+++
Clubbed tips (CT)	−	+	+	++
Degeneration in primary lamellae (DPL)	−	++	+	+++

*Intestine abnormalities*				
Epithelium breakage (EB)	−	++	+	++
Enterocyte vacuolization (EV)	−	++	++	+++
Epithelial detachment from the lamina propria (DEL)	−	+++	+	+++
Fusion of brush border (F)	−	++	−	+++
Villus beheading (BV)	−	++	+	+++
Vacuolization in submucosa (VSM)	−	++	+	+++

*Liver abnormalities*				
Degeneration of bile duct (DBD)	−	−	++	+++
Blood congestion (BC)	−	−	++	++
Hemorrhage (H)	−	+	+	+++
Necrosis (N)	−	−	+	++
Patchy degeneration (PD)	−	−	++	+++
Hypertrophy nucleus (HN)	−	−	−	++
Melanomacrophage centers (MMCs)	−	−	++	+++
Hepatocyte vacuolation (HV)	+	+	++	+++

*Kidney abnormalities*				
Blood congestion (BC)	−	−	++	++
Increase in the diameter of renal tubules (RT)	−	−	++	+++
Dilation of Bowman’s capsule (DB)	−	+	+	++
Necrosis (N)	−	−	+	++
Vacuolation (V)	−	−	+++	+++
Melanomacrophage center (MMC)	−	−	−	++

*Note:* MP: microplastic; Hg: mercury (Hg); −, none (0%); +, mild (< 10%); ++, moderate (10%–50%); +++, severe (> 50%).

#### 3.5.2. Structural Changes in the Intestine

**FIGURE 5:**
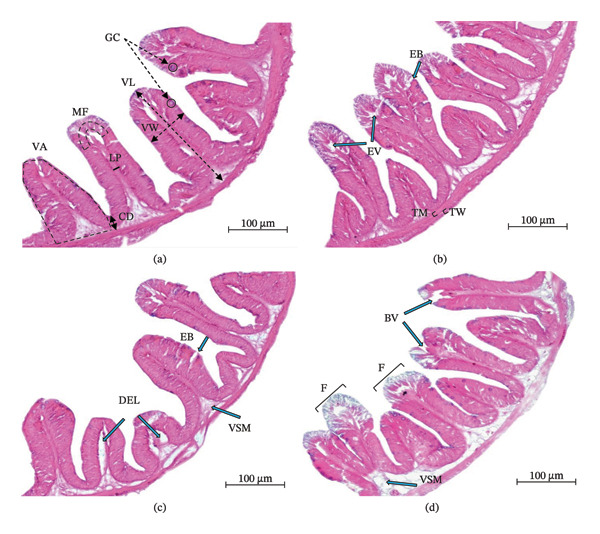
The histoarchitectural alterations in the intestine of Nile tilapia exposed to MP and/or Hg: (a) morphometric parameters (VL: villus length; VW: villus width; VA: villus area; CD: crypt depth; LP: width of lamina propria; MF: fattening of mucosal fold; GC: goblet cell; TM: thickness of muscular; TW: thickness of the wall); (b–d) abnormalities: epithelium breakage (EB), enterocyte vacuolization (EV), epithelial detachment from the lamina propria (DEL), fusion of brush border (F), villus beheading (BV), vacuolization in submucosa (VSM); MP: microplastic; Hg: mercury.

**FIGURE 6:**
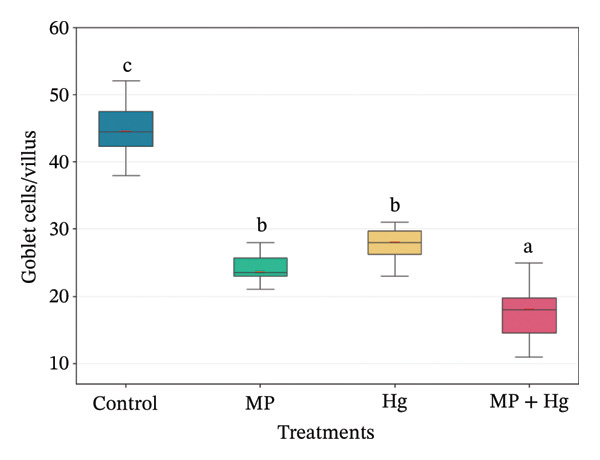
Intestinal cellular composition (goblet cells) in Nile tilapia exposed to MP and Hg treatments for 42 days; data are expressed as mean ± standard deviation (SD); superscripts (a, b, and c) indicate significant variation (*p* < 0.05) among treatments; MP: microplastic; Hg: mercury.

Abnormalities, including epithelium breakage, enterocyte vacuolization, epithelial detachment from the lamina propria, brush border fusion, villus beheading, and submucosal vacuolization, were observed in the intestine (Figure 5). The intestinal tissue of Nile tilapia (*O. niloticus*) in the control group appeared intact with no detectable histopathological alterations. As indicated in Table 4, the semiquantitative assessment of intestinal tissue in the MP + Hg exposure group and MP group showed pronounced pathological severity relative to the corresponding group exposed solely to Hg. Furthermore, intestinal cellular composition (goblet cells) showed lower counts (*p* < 0.05; Figure 6) in the MP and MP + Hg groups compared with the untreated condition.

#### 3.5.3. Structural Changes in the Liver

**FIGURE 7:**
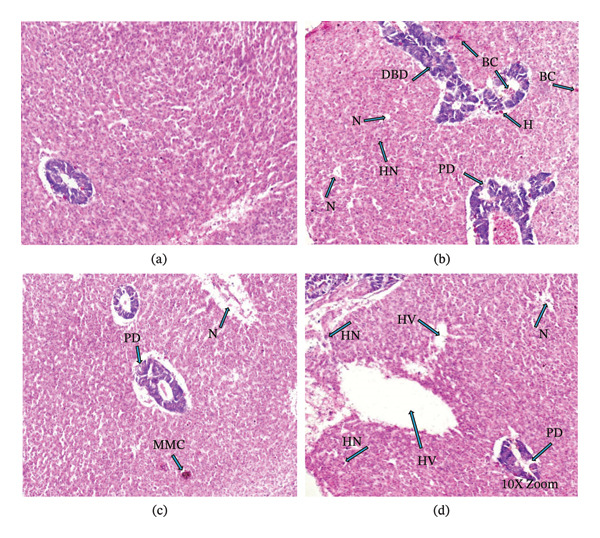
The histoarchitectural alterations in the liver of Nile tilapia exposed to MP and/or Hg: (a) control; (b–d) alterations: degeneration of bile duct (DBD), blood congestion (BC), hemorrhage (H), necrosis (N), patchy degeneration (PD), hypertrophy nucleus (HN), melanomacrophage centers (MMCs), hepatocyte vacuolation (HV); MP: microplastic; Hg: mercury.

The liver of Nile tilapia (*O. niloticus*) fingerlings was also damaged by the inclusion of MP and Hg and exerted mild‐to‐moderate histoarchitectural deviations (Table 4). Profound structural changes in the liver included bile duct degeneration, blood congestion, hemorrhage, necrosis, patchy degeneration, hypertrophy nucleus, melanomacrophage centers, and hepatocyte vacuolation (Figure 7b, c, d). In our study, histological examination indicated that in the control group, the liver of fingerlings showed regular morphology and systematic cellular arrangement and maintained an intact cytomembrane (Figure 7a). However, the livers of fish in the Hg and MP + Hg groups were irregularly shaped and showed moderate (10%–50%) to severe (> 50%) degeneration of the bile duct, blood congestion, patchy degeneration, melanomacrophage centers, and vacuolation. In the MP exposure group, the liver showed mild (< 10%) alterations, such as hemorrhage and vacuolation.

#### 3.5.4. Structural Changes in the Kidney

**FIGURE 8:**
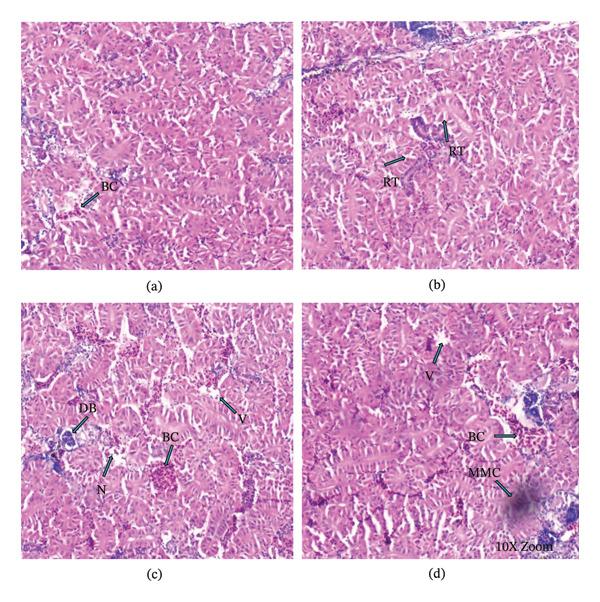
(a–d) Structural abnormalities in histoarchitecture of kidney Nile tilapia exposed to MP and/or Hg; blood congestion (BC), increase in the diameter of renal tubules (RT), dilation of Bowman’s capsule (DB), necrosis (N), vacuolation (V), melanomacrophage centers (MMCs); MP: microplastic; Hg: mercury.

Under light microscopy, the kidney tissue of Nile tilapia (*O. niloticus*) in the control and MP groups displayed intact structures, except for mild (< 10%) dilation of Bowman’s capsule in the MP‐treated group. However, abnormalities such as blood congestion, renal tubular dilation, dilation of Bowman’s capsule, necrosis, vacuolation, and melanomacrophage center (Figure 8) were observed in the kidney of fingerlings exposed to Hg and MP + Hg. Semiquantitative assessment indicated that the kidney of fish in the Hg and MP + Hg groups was irregularly shaped and showed moderate (10−50%) to severe (> 50%) degeneration (Table 4).

### 3.6. Transcriptional Responses of gh in the Pituitary and igf‐1 and igf‐2 in the Liver

**FIGURE 9:**
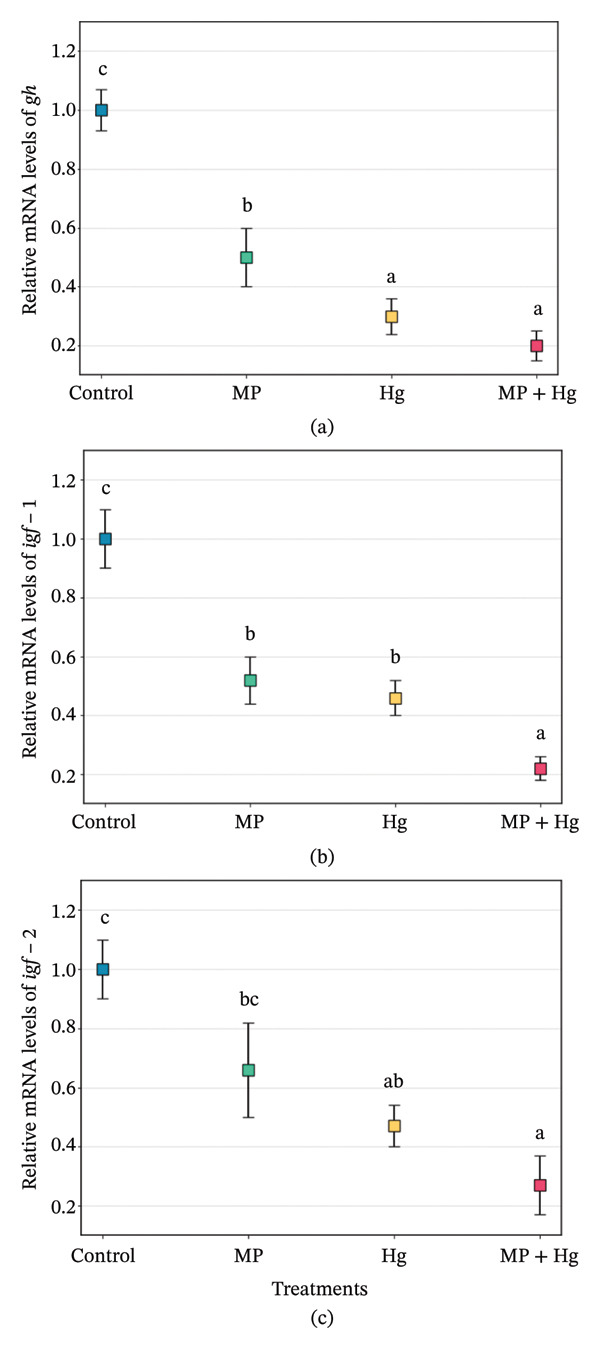
Expression profiles of growth‐related mRNAs: (a) growth hormone (*gh*), insulin‐like growth factors (b) *igf-1* and (c) *igf-2* in Nile tilapia exposed to MP and Hg treatments for 42 days; superscripts (a, ab, bc, b, and c) indicate significant variation (*p* < 0.05) among treatments; MP: microplastic; Hg: mercury.

The transcriptional profiles of growth hormone (*gh*) in the pituitary gland and insulin‐like growth factors (*igf-1* and *igf-2*) in the liver of Nile tilapia (*O. niloticus*) under different treatments are presented in Figure 9. In the control group, mRNA expression patterns of *igf-1* reached their highest level and showed a progressive downward shift (*p* < 0.05) following exposure to MP, Hg, and MP + Hg treatments, but no significant difference (*p* > 0.05) was detected between the MP and Hg groups. In the case of *igf-2*, the lowest expression was observed in the MP + Hg, which was significantly lower (*p* < 0.05) than that of the control group, indicating a strong suppressive effect of combined exposure. The expression levels of *gh* in the pituitary gland showed a progressive downward trend (*p* < 0.05) under all test conditions, with the lowest level in MP + Hg, but no significant difference (*p* > 0.05) was detected between the Hg and MP + Hg groups.

### 3.7. Transcriptional Responses of cat and Superoxide Dismutase (sod) in the Liver

**FIGURE 10:**
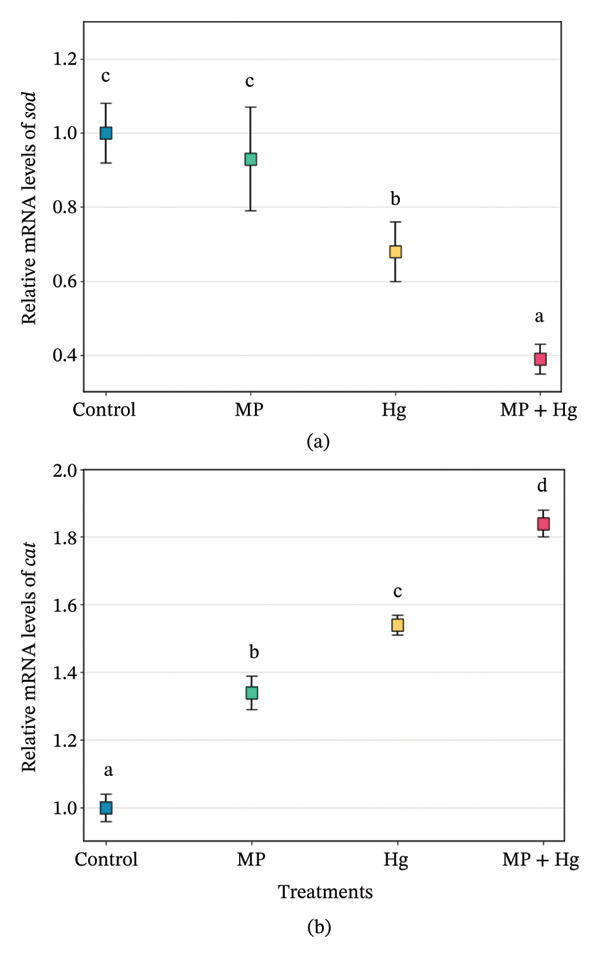
Expression profiles of antioxidant‐related mRNAs: (a) superoxide dismutase (*sod*) and (b) catalase (*cat*) in the liver of Nile tilapia exposed to MP and Hg treatments for 42 days; superscripts (a, b, c, and d) indicate significant variation (*p* < 0.05) among treatments; MP: microplastic; Hg: mercury.

The hepatic transcriptional activities of *sod* and catalase (*cat*) as evidenced by their mRNA levels are illustrated in Figure 10. In the control group, gene expression patterns of *cat* reached their lowest level and showed a progressive upward trend (*p* < 0.05) following exposure to MP, Hg, and MP + Hg. An opposite shift was observed for *sod* expression, which was highest in the control group and exhibited a downward modulation (*p* < 0.05) under test conditions, reaching its lowest level in the MP + Hg group; however, no significant difference (*p* > 0.05) was detected between the control and MP groups.

### 3.8. Transcriptional Responses of il‐1β, ifn‐γ, and tnf‐α in the Liver

**FIGURE 11:**
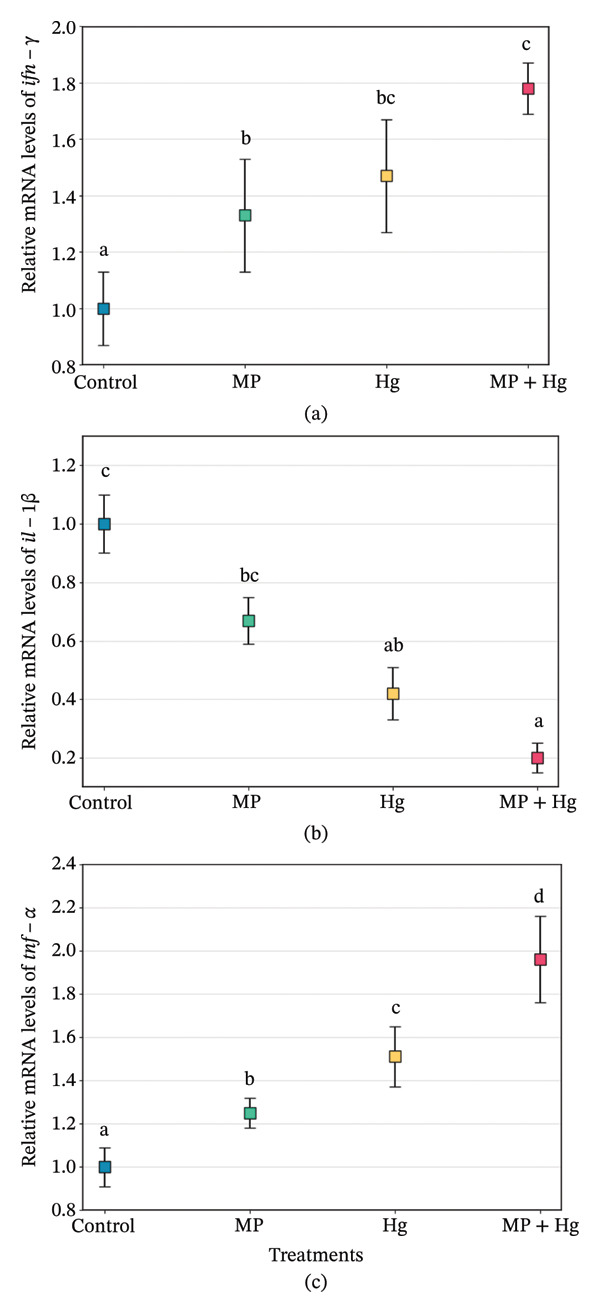
Expression profiles of immune‐related mRNAs: (a) interferon‐*γ* (*ifn-γ*), (b) interleukin‐1β (*il-1β*), and (c) tumor necrosis factor‐*α* (*tnf-α*) in the liver of Nile tilapia exposed to MP and Hg treatments for 42 days; superscripts (a, ab, bc, b, c, and d) indicate significant variation (*p* < 0.05) among treatments; MP: microplastic; Hg: mercury.

Figure 11 presents the transcriptional profiles of interferon‐*γ* (*ifn-γ*) and tumor necrosis factor‐*α* (*tnf-α*) and interleukin‐1 *β* (*il-1β*), in the liver of Nile tilapia (*O. niloticus*) subjected to different treatment conditions. The expression level of *il-1β* was elevated in the control group and showed a downward trend (*p* < 0.05) under the test conditions, with the lowest level observed in the MP + Hg group; however, no significant difference (*p* > 0.05) was found in *il-1β* expression between the MP and Hg groups. An inverse shift was observed for *ifn-γ* expression, which was lower in the control group and showed an upward trend (*p* < 0.05) under test conditions, reaching its highest level in the MP + Hg group, but no significant difference (*p* > 0.05) was noted between the MP and Hg groups. The expression levels of *tnf-α* in the liver showed a progressive upward trend (*p* < 0.05) under the test conditions, reaching its highest level in the MP + Hg group.

### 3.9. PCA

**FIGURE 12:**
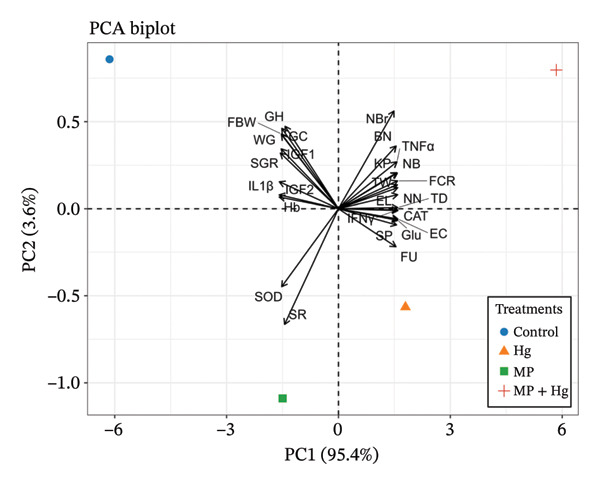
Biplot of principal component analysis (PCA) using several parameters of Nile tilapia (MP: microplastic; Hg: mercury; FCR: feed conversion efficiency; WG: weight gain; FBW: final body weight; SGR: specific growth rate; SR: survival rate; Glu: glucose; Hb: hemoglobin; GC = goblet cells; KP: karyopyknosis; BN: binucleated; NN: notched nuclei; NBr: nuclear bridge; NB: nuclear bud; TW: twin; FU: fusion; TD: teardrop; SP: spindle; EL: elongated; EC: echinocyte).

The PCA biplot explained 99.0% of the total variance (PC1: 95.4% and PC2: 3.6%), effectively separating the test conditions based on growth indices, hematological parameters, and antioxidant–immune responses (Figure 12). The control group clustered on the far left of PC1 and was positively associated with growth indices (FBW, WG, SGR, FCR, and GC), endocrine markers (*igf-1*, *igf-2*, and *gh*), and hemoglobin (Hb), indicating an optimal physiological condition under normal conditions. The MP group was positioned in the lower‐left quadrant, showing moderate separation from the control and negative correlations with survival rate (SR) and *sod*, suggesting mild physiological stress and early activation of antioxidant responses. In contrast, the Hg‐treated group appeared in the lower‐right quadrant, closely aligned with immune‐related genes (*tnf-α* and *il-1β*) and stress markers, indicating inflammation and moderate physiological impairment. The MP + Hg group was located in the upper‐right quadrant and strongly linked to oxidative stress indicators (*cat* and *sod*), immune activation, and reduced growth metrics, demonstrating the most pronounced physiological disruption under combined exposure. These results suggest that both MP and Hg individually impair the physiology of Nile tilapia (*O. niloticus*), whereas coexposure leads to the greatest disturbances across growth, hematological indices, and antioxidant–immune gene profiles.

## 4. Discussion

Aquatic pollution endures as a high‐priority ecological issue globally due to its widespread detrimental impacts on aquatic organisms from contaminants such as MPs [1, 2], HMs [32], pesticides [39], and industrial effluents [40]. Among these, the occurrence of MPs in freshwater ecosystems is emerging as a global issue due to their escalating prevalence and low degradation potential [41]. In aquatic environments, MPs may absorb multiple pollutants, thus potentially acting as a vector of these pollutants (also known as the vector effect) [42], and the interrelation of MPs with multiple pollutants may exert combined effects that increase systemic stress in aquatic animals [4, 43]. To address this, we executed a 42‐day standardized lab‐based trial to investigate the concurrent effects of PA‐MP and Hg on the physiology of Nile tilapia (*O. niloticus*).

Fish subjected to each PA‐MP exposure condition ingested MP particles, with significantly greater quantities detected in the GIT and gills of Nile tilapia under the MP + Hg condition than those in the MP‐only group, indicating that Hg exposure enhanced MP uptake. This effect may arise from Hg, which enhances overall metabolic activity by impeding the acetylcholinesterase (AChE) enzyme and initiating the secretion of stress hormones such as cortisol [44, 45]. This rise in metabolism may stimulate appetite and foraging behavior, causing fish to increase their intake and unintentionally ingest MPs that appear similar to natural food [6, 46]. Shahriar et al. [4] reported that coexposure to fenitrothion can enhance MP ingestion in juvenile striped catfish (*Pangasianodon hypophthalmus*), a pattern consistent with our findings. Likewise, coexposure to Pb and MPs in the mangrove crab (*Minuca vocator*) has shown a synergistic effect, with significantly elevated tissue bioaccumulation and oxidative stress [47].

Growth is recognized as an important physiological indicator that integrates numerous biological mechanisms and metabolic functions in an organism. In this investigation, a notable reduction in growth, as evidenced by FBW, WGR, and SGR, occurred after both individual exposure and combined exposure to MP and Hg. This finding suggests an aggravation of growth suppression when MPs occurred concurrently with Hg. Islam et al. [48] reported that the concurrent exposure to Cr and PA‐MP over 42 days caused pronounced reductions in growth performance in Nile tilapia. Reports suggest that prolonged exposure to HMs (e.g., Cr and Cu) impairs growth and survival by disrupting metabolic pathways, distorting enzymatic homeostasis, and elevating oxidative stress [43, 48]. Conversely, MPs can accumulate on the intestinal walls, narrowing the intestinal lumen and obstructing the passage of food due to their small size and lipophilic attributes [4]. This can also suppress enzyme secretion and impair digestion and absorption of vital nutrients, which may result in suboptimal growth and compromised physiological performance [6]. Likewise, micro‐ and/or nanoscale plastic debris (< 1 μm) has the capacity to enter the vascular system, triggering oxidative stress, and thus attenuates growth and feed assimilation, as well as raises mortality in fish [49]. Reports suggest that MPs act as carriers when combined with Hg, enhancing Hg accumulation and toxicity, which, in turn, impairs growth and development in marine copepods and zebrafish (*Danio rerio*) through induction of oxidative stress, apoptosis, and metabolic dysfunction [50, 51].

Blood, as a fluid connective tissue, represents one of the most important biological media within the body. Its composition undergoes alterations in response to environmental pollutants, reflecting disruptions in key physiological and biochemical mechanisms and altering homeostatic balance in fish [52]. Blood biomarkers, including glucose and hemoglobin concentrations, as well as erythrocytic morphological anomalies, are useful indicators, as these parameters show a rapid reaction if fishes are exposed to contaminants and/or stressful conditions [53]. Reports have shown that exposure to either MPs or HMs solely causes stress in fish by shifting serum biochemical composition [48, 54]. For instance, HM toxicity has been reported to induce hyperglycemia (elevated glucose levels) and hypohemoglobinemia (reduced hemoglobin levels) in freshwater fish species, including African catfish (*Clarias gariepinus*), Nile tilapia (*O. niloticus*), and zebrafish (*D. rerio*) [54, 55]. Likewise, MP‐induced stress exerted adverse effects on serum biochemical profiles in striped catfish and Nile tilapia [4, 56]. In this investigation, exposure to PA‐MP and Hg, when administered either individually or concurrently, resulted in an elevation in serum glucose levels alongside a reduction in hemoglobin concentrations, indicating a clear physiological stress response. Elevated glucose levels may result from hepatic glycogenolysis (glycogen breakdown in the liver), disruption of the glucose transport mechanism, or a compensatory response to energy deficiency in fish exposed to environmental contaminants [32, 39]. The hypothalamic–pituitary–interrenal (HPI) axis modulates this metabolic shift by stimulating cortisol release and enhancing energy reserve utilization, thereby raising plasma glucose concentrations to satisfy heightened energy requirements [57]. In addition to glucose regulation, hemoglobin reflects physiological stress and is a key serum parameter involved in oxygen transport; its decline indicates anemia, which can impair oxygen delivery and affect metabolic and physiological health in fish [53]. The toxicity of MP and HM includes a decline in blood hemoglobin concentrations, serving as a clear indicator of anemia in fish, often resulting from iron deficiency and leading to hypoxia due to decreased oxygen‐binding capacity [13, 52, 58].

Erythrocytes, or red blood cells (RBCs), are profoundly sensitive in response to chemical toxicants or environmental stressors, responding immediately by generating aberrant fractures in their cellular morphology (ECA) and/or nuclear structure (ENA) [53]. Environmental contaminants such as MPs and HMs can disrupt erythrocyte structure in fish, thereby causing shrinkage, deformation, and impaired oxygen transport efficiency [32, 53]. Structural changes, particularly at the cellular and membrane levels, can serve as sensitive early indicators of genotoxicity caused by toxins [59], through either mutation induction or chromosome fragmentation before mitosis [60]. In our study, concurrent exposure escalated the prevalence of nuclear deformities, including karyopyknosis, binucleated, notched nuclei, nuclear bridges, and nuclear buds. The high prevalence of ENAs suggests that exposure to MP and Hg induces genotoxicity, most likely via chromosomal instability, nuclear envelope remodeling, cytoskeletal dysfunction, and impaired mitotic progression [60, 61]. However, the most prevalent ECAs in the MP + Hg group were twin, fusion, teardrop, spindle, elongated, and echinocyte. Earlier investigations have reported that the assimilation, accumulation, and toxicological effects of hazardous substances trigger oxidative stress, compromise cellular membranes and organelles, and impair enzymatic activity, cumulatively inducing erythrocytic deformities in fish [13, 62].

Histopathological approaches in fish toxicology provide indispensable information on how toxic substances interfere with physiological processes. Studies have demonstrated that acute and/or chronic exposure of MPs, pesticides, and HMs exerts significant structural deviations in the gills, intestine, liver, and kidney of fish [52, 63]. Although histopathological alterations resulting from MP exposure in fish have been extensively studied, limited information is known about the effects of Hg, either individually or in combination with MPs, on various internal organs of fish. Considering the toxic effects of MP and Hg individually, we hypothesize that combined exposure to MP and Hg may exert more pronounced tissue damage than single exposure, due to their cumulative toxicological effects.

Gills are an important organ associated with gaseous exchange and ionic equilibrium between the fish body and its surrounding environment. Due to their direct exposure to external conditions, they serve as the main pathway for contaminant uptake [64]. Being highly sensitive to environmental contaminants, gills are prone to higher contaminant exposure and endure the most severe damage. In our study, pronounced gill tissue damage was evident, including epithelial lifting, lamellar fusion, hyperplasia, telangiectasia, congestion in primary lamellae, deformed pillar system, clubbed tips, and degeneration within the primary lamellae, following MP and MP + Hg exposure. These gill tissue deformities are consistent with findings from ecotoxicological studies in which various fish species were exposed to MPs and HMs or their combined exposure [17, 65, 66]. Kim et al. [65] reported that blood cell counts, along with hypertrophy, hyperplasia, and terminal clubbing of the gill lamellae, were evident in zebrafish (*D. rerio*) after 14 days of exposure to the combined toxicity of PA‐MP and Cu. Reports showed that gill dysfunction in fish caused by HM exposure is associated with vascular injury, cytoskeletal disruption, ionoregulatory disruption, and impaired respiration [67, 68]. However, small MPs aggregating within the gill tissue may cause clogging and disrupt osmoregulation, whereas irregularly shaped particles with abrasive edges can inflict physical injuries that impair the normal structure and functional capacity of the gill [27]. We speculate that physical injuries may facilitate the entry of smaller MPs and Hg into the bloodstream, thereby escalating their genotoxic and cytotoxic effects.

Fish intestines act as an exposure route for various pollutants that pass into the body via water or food and play a pivotal role in their absorption and metabolic processing [52]. However, the uptake of toxic substances through gastrointestinal membranes can cause multiple forms of damage; therefore, evaluating intestinal health may offer valuable evidence of systemic anomalies exerted by various toxins. This investigation found severe inflammatory responses in the MP and MP + Hg test conditions, including epithelium breakage, enterocyte vacuolization, epithelial detachment from the lamina propria, brush border fusion, villus beheading, and submucosal vacuolization. The observed inflammatory responses in intestinal tissue align with results from aquatic toxicology studies where fish were subjected to MP, HM, or a combination of both [48, 63, 65]. Islam et al. [48] identified epithelial detachment, villi beheading, fusion, hyperplasia, and swelling of intestinal villi in Nile tilapia after 42 days of combined exposure to PA‐MP and Cr. Toxic substances such as MPs and HMs are easily absorbed onto the surface of intestinal villi in fish and enhance cell membrane permeability through interactions with membrane phospholipids [63]. These substances damaged the surface of the intestine and triggered a cascade of inflammatory responses [69, 70]. In addition, intestinal cellular components such as goblet cells play a significant role in the secretion of mucin, which lubricates and protects the intestinal surface. However, pollutants such as MPs and HMs can damage these cells, leading to swelling of goblet cells and a reduction in their abundance [11, 52], which aligns with our findings. We hypothesize that sharp‐edged brush filaments may have caused injuries in the intestine of Nile tilapia, which allow MP and Hg to enter the bloodstream and intensify their toxic effects.

Fish liver is an important organ because it has been regarded not only as the main toxicant‐deposition site for xenobiotic agents but also as an active participant in their detoxification process [7]. The accumulation, biotransformation, and metabolism of xenobiotics can exert hepatotoxicity, which is ultimately reflected in various histoarchitectural changes in the liver. In this investigation, we found severe hepatic damage following Hg and MP + Hg exposure, including degeneration of the bile duct, blood congestion, hemorrhage, necrosis, patchy degeneration, hypertrophy nucleus, melanomacrophage centers, and hepatocyte vacuolation. Reports showed that other HMs or MPs, either separately or jointly, elicit similar hepatic tissue alterations in various freshwater fish species [71, 72]. Islam et al. [48] identified necrosis, MMC, vacuolation, and patchy degeneration in the liver of Nile tilapia after 42 days of combined exposure to PA‐MP and Cr. Chronic exposure of sublethal Hg (1/10th of LC_50_) imparted histopathological changes in the hepatic tissue of zebrafish (*D. rerio*) due to the lipidomic imbalance, glycogen depletion, hepatocyte apoptosis, and DNA damage, which collectively indicate the potential consequences of Hg toxicity [72]. In addition, MP‐mediated hepatic impairment in fish is linked to capillary congestion, mitochondrial dysfunction, generation of reactive oxygen species (ROS), and dysregulated protein synthesis [73].

The kidney is a fundamental organ that filters blood and facilitates the excretion of toxic metabolites and xenobiotics from the body [22]. Therefore, the presence of lesions in renal tissue may be considered a reliable indicator of aquatic pollution. In the present study, exposure to PA‐MP and Hg observed several histopathological deviations in kidney structure, including blood congestion, renal tubular dilation, dilation of the Bowman’s capsule, necrosis, vacuolation, and melanomacrophage centers in Nile tilapia fingerlings, which are analogous to the findings of established scientific reports [48, 65, 74]. Radi [75] reported that chronic exposure to systemic pollutants elevates the risk of oxidative stress, morphological damage, and functional impairment. MPs function as vectors for HMs and increase their accumulation in fish kidneys by elevating bioavailability and enabling uptake [76]. Their strong affinity for metal binding may extend Hg retention and enhance its absorption. A study reported that once ingested, MPs interfere with renal clearance, obstruct copper excretion, initiate oxidative stress, and alter metal‐binding proteins such as metallothioneins, collectively escalating copper persistence and toxicity in aquatic biota [77].

Growth is mainly mediated by the *gh* and/or insulin‐like growth factor axis, which is highly conserved in fish. The GH/IGF axis becomes activated when the hypothalamus secretes GHRH, which stimulates the anterior PG to synthesize and release *gh* [78]. *gh* binds to its receptor (GHR) and subsequently increases the expression of insulin‐like growth factors in the liver to regulate somatic growth in fish [79]. The circulating IGFs are recognized as reliable biomarkers of accelerated growth in fish, as they interact with IGFRs to mediate growth‐promoting signals [80]. IGF‐binding proteins (IGFBPs) are essential modulators of IGF, contributing to their stabilization and systemic transport, and are involved in regulating a diverse physiological process such as growth, development, and metabolism [81]. However, environmental pollutants can disrupt the GH/IGF axis by altering hormone synthesis, receptor sensitivity, and downstream signaling pathways, compromising growth and metabolic homeostasis in fish [3, 82]. In this study, expression levels of *igf-1*, *igf-2*, and *gh* showed a downward modulation following exposure to MP, Hg, and MP + Hg treatments, indicating a disruption of the GH/IGF axis. Consistently, Chen et al. [83] reported a downregulation of *igf-1* and *gh* in zebrafish (*D. rerio*) exposed to both MP and Cd, either alone or in combination. Recent reports indicate that MPs exert transgenerational effects on key regulatory pathways such as *nrf2* signaling cascade, GH/IGF axis, and HPI axis [78], which may indirectly affect the transcriptional regulation of growth‐associated genes. Besides, HMs exert oxidative stress through ROS production and compromise antioxidant defense, thereby disrupting mitochondrial function and redox‐sensitive transcription factors [5], which may suppress the expression of growth genes. Canosa et al. [82] reported that chronic HM stress activates the HPI axis, resulting in elevated cortisol levels that exert glucocorticoid‐mediated inhibition on *gh* secretion and subsequent IGF production.

Environmental contaminants can trigger oxidative stress in animals, characterized by the overproduction of ROS and free radicals, which surpass the inherent defense mechanisms and compromise cell integrity. Antioxidant genes like *sod* and *cat* are key enzymatic markers of oxidative stress and are involved in the first line of defense against ROS. *sod* enables the transformation of superoxide radicals (O_2_) into hydrogen peroxide (H_2_O_2_), which is later converted into water and oxygen by *cat* [84]. Scientific evidence indicates that MPs and HMs, whether acting independently or in combination, exert oxidative stress by disrupting the activities of *sod* and *cat*. In our study, expression levels of *sod* showed a downward modulation, potentially reflecting enzyme inhibition or exhaustion, whereas *cat* showed an upward modulation in the liver following exposure to the MP, Hg, and MP + Hg treatments, indicating the onset of oxidative stress. Previous reports indicate that extended exposure to MPs leads to elevated *cat* levels and suppressed *sod* levels in Nile tilapia [56, 85]; similarly, Cd exposure has been reported to elevate *cat* levels in largemouth bass (*Micropterus salmoides*) and suppress *sod* levels in snakehead (*Channa argus*) [86, 87]. Under coexposure to MPs and HMs, reports have shown similar modulation patterns of *cat* and *sod* in northern snakehead (*C*. *argus*) species [66]. The toxic effects of MP and HM in fish are primarily mediated through the overproduction of ROS, which disrupts cellular redox homeostasis and exerts oxidative damage such as lipid peroxidation (LPO), thereby impairing membrane integrity and cell function [32, 88]. However, the increased activity of *cat* reflects an adaptive response to counteract oxidative damage and maintain cellular integrity [89].

Proinflammatory cytokines such as *il-1β*, *tnf-α*, and *ifn-γ* play pivotal roles in fish immune regulation, with *il-1β* mediating inflammatory signaling, cell proliferation, and immune metabolism; *tnf-α* modulating immunocyte functions; and *ifn-γ* enhancing both innate and adaptive immunity through macrophage activation and antigen presentation [90, 91]. However, exposure to toxic agents may provoke an inflammatory response in fish through the modulation of these genes, thereby intensifying immune regulation. Reports have demonstrated that HMs can exert cytotoxic and genotoxic effects by damaging genomic DNA through their accumulation in internal tissues of fish [22, 66]. Besides, exposure to relatively smaller MPs is presumed to induce immunotoxicity by disrupting the immune response and modulating cytokine expression [6]. The mRNA level of *il-1β* showed downward modulation in Nile tilapia exposed to MP and Hg, either alone or in combination, in this investigation. Consistently, Wang et al. [86] reported downregulation of *il-1β* in hybrid snakehead exposed to both MP and Cd, either alone or combined. In general, *il-1β* is upregulated during immune activation, but it may be downregulated under prolonged exposure to multiple stressors due to glucocorticoid‐mediated immunosuppression and epigenetic modifications such as DNA methylation and histone modification [92]. This downward modulation indicates a weakened inflammatory response, which may undermine immune defense mechanisms. Conversely, this study experienced an upward modulation in the expression of *tnf-α* and *ifn-γ* in Nile tilapia exposed to MP and Hg, whether administered individually or concurrently. Scientific evidence indicates that *tnf-α* was upregulated in crucian carp (*Carassius carassius*) and Prussian carp (*C*. *gibelio*) under Cd exposure [7], whereas both *tnf-α* and *ifn-γ* were upregulated in Nile tilapia under Cu exposure [24]. Besides, Haque et al. [56] reported that exposure to PA‐MP for 42 days elicited a noticeable transcriptional upregulation of *tnf-α* and *ifn-γ* in Nile tilapia. Under coexposure to MPs and HMs, reports have shown similar upward modulation expression patterns of *tnf-α* and *ifn-γ* in Nile tilapia [48]. Evidence indicates that MPs act as carriers for HMs and increase toxicity through synergistic interaction by increasing their bioavailability and facilitating tissue accumulation [42]. This coexposure stimulates overproduction of ROS, disrupts membrane integrity, and activates inflammatory signaling pathways such as *nf-κb* and *mapk*; these processes ultimately lead to oxidative stress and immune dysregulation in fish [93–95].

The coexistence of MPs and Hg in environmental compartments often creates concurrent exposure routes for organisms, which increases ecological concern. When MPs and Hg are jointly present, their interaction may modify their individual toxicities, which enhances the combined toxic effects and indicates cumulative chemical impacts, as MPs act as vectors for environmental contaminants [42]. Our experimental outcomes suggest that simultaneous exposure to multiple pollutants (e.g., MPs and Hg) can aggravate systemic and molecular impairments, underscoring the complexity of contaminant interactions in real‐world aquatic environments. The implications of these findings are significant for aquaculture practices and environmental management strategies. First, the accumulation of MPs in tissues, coupled with disrupted growth and physiological functions, signals risks to fish health and suggests that conventional monitoring may underestimate hazards of co‐occurring contaminants. Second, alterations in the GH/IGF axis and immune–antioxidant responses suggest that chronic exposure could compromise growth efficiency and immune competence, with far‐reaching impacts on food safety and environmental sustainability. However, this experiment was limited to a 42‐day exposure period with fixed concentrations of 10 mg/L MP and 0.03 mg/L Hg, which may not fully capture the variability of environmental levels or the effects of chronic low‐dose exposures in natural ecosystems. Moreover, the study examined only a single fish species and life stage, which restricts the generalizability of the findings. The endpoints assessed were confined to selected physiological, histological, endocrine, immune, and antioxidant parameters, whereas other relevant biomarkers such as gut microbial activity, enzymatic responses, and reproductive performance were not included. Future investigations should address long‐term, environmentally relevant exposure scenarios across diverse species and life stages, incorporate a wider spectrum of molecular, reproductive, and microbiome biomarkers, and examine interactions with additional co‐occurring pollutants to strengthen ecological risk assessments and promote sustainable aquaculture practices.

## 5. Conclusion

This investigation revealed that the toxicity of PA‐MP along with the presence of Hg significantly escalated MP uptake, reduced survivability, and adversely altered the blood chemistry, internal organ histopathology and antioxidant, and immune and growth‐related genetic responses in Nile tilapia. Besides, this issue raises serious concern, as Hg is well‐established to be bioaccumulative and biomagnifying in fish, whereas MPs are bioaccumulative, but their biomagnification potential remains debated and requires further clarification. These contaminants may enter the human food chain and pose health risks. Unfortunately, the concentrations of these hazardous substances continue to rise in the natural environment due to indiscriminate waste disposal into the open environment. Therefore, effective waste disposal strategies should be implemented globally to safeguard environmental health and minimize toxicity. Future investigations should focus on long‐term field studies to assess the effects of different sizes and shapes of MPs, their coexposure with other HMs, and interactions with other key environmental pollutants to evaluate real‐world coexposure toxicological paradigms in natural aquatic environments.

## Author Contributions

Md Ruhul Amin: writing–reviewing and editing, writing–original draft, visualization, formal analysis, data curation, investigation, and conceptualization; Moshfiq Momtasir Neloy and Tasmia Islam Kanta: data curation and investigation; Md. Mahiuddin Zahangir: writing–reviewing and editing and resources; Saleha Khan: writing–reviewing and editing; and Md Shahjahan: writing–reviewing and editing, supervision, fund acquisition, and conceptualization.

## Funding

No funding was obtained for this manuscript.

## Conflicts of Interest

The authors declare no conflicts of interest.

## Data Availability

Data sharing is not applicable to this article as no datasets were generated or analyzed during the current study.

## References

[1] Sun Q. , Ren S.-Y. , and Ni H.-G. , Incidence of Microplastics in Personal Care Products: An Appreciable Part of Plastic Pollution, Science of the Total Environment. (2020) 742, 10.1016/j.scitotenv.2020.140218. 32629242

[2] Al-Emran M. , Sarker A. , Adib S. S. et al., Microplastics Pollution in Aquatic Ecosystems of Bangladesh—A Critical Review on Research Trends and Future Perspectives, Science of the Total Environment. (2025) 1002, 10.1016/j.scitotenv.2025.180620. 41027357

[3] Ghasemi A. and Shadi A. , Combined Effects of Microplastics and Benzo[A]Pyrene on Asian Sea Bass *Lates calcarifer* Growth and Expression of Functional Genes, Comparative Biochemistry and Physiology, Part C: Toxicology & Pharmacology. (2024) 283, 10.1016/j.cbpc.2024.109966. 38897364

[4] Shahriar S. I. M. , Islam N. , Emon F. J. , Nepal V. , Khan S. , and Shahjahan M. , Combined Impacts of Organophosphate Pesticide and Polyamide Microplastics on Growth, Hematology, and Immune Responses in Juvenile Striped Catfish (*Pangasianodon hypophthalmus*), Emerging Contaminants. (2025) 11, no. 3, 10.1016/j.emcon.2025.100520.

[5] Pu Y. , Guo J. , Yang H. et al., Environmentally Relevant Concentrations of Mercury Inhibit the Growth of Juvenile Silver Carp (*Hypophthalmichthys molitrix*): Oxidative Stress and GH/IGF Axis, Ecotoxicology and Environmental Safety. (2022) 236, 10.1016/j.ecoenv.2022.113484. 35421826

[6] Kim J.-H. , Yu Y.-B. , and Choi J.-H. , Toxic Effects on Bioaccumulation, Hematological Parameters, Oxidative Stress, Immune Responses and Neurotoxicity in Fish Exposed to Microplastics: A Review, Journal of Hazardous Materials. (2021) 413, 10.1016/j.jhazmat.2021.125423. 33930961

[7] Wei W. , Yang Q. , Xiang D. et al., Combined Impacts of Microplastics and Cadmium on the Liver Function, Immune Response, and Intestinal Microbiota of Crucian Carp (*Carassius carassius*), Ecotoxicology and Environmental Safety. (2023) 261, 10.1016/j.ecoenv.2023.115104. 37295303

[8] Briffa J. , Sinagra E. , and Blundell R. , Heavy Metal Pollution in the Environment and Their Toxicological Effects on Humans, Heliyon. (2020) 6, no. 9, 10.1016/j.heliyon.2020.e04691. PMC7490536 32964150

[9] Veisi R. S. , Taghdir M. , Abbaszadeh S. , and Hedayati A. , Dietary Effects of Probiotic *Lactobacillus casei* on Some Immunity Indices of Common Carp (*Cyprinus carpio*) Exposed to Cadmium, Biological Trace Element Research. (2023) 201, no. 2, 959–967, 10.1007/s12011-022-03205-7. 35325365

[10] Badr A. , Haverinen J. , and Vornanen M. , Effects of Inorganic Mercury (HgCl_2_) on Electrical Excitability of Rainbow Trout (*Oncorhynchus mykiss*) Heart, Environmental Toxicology and Chemistry. (2025) 44, no. 8, 2206–2220, 10.1093/etojnl/vgaf128. 40392210

[11] Chu T. , Xu B. , Guo F. , Zhu M. , and Yang R. , Co-Exposure to Polystyrene Nanoplastics and Mercury Synergistically Exacerbates Toxicity in Rare Minnow (*Gobiocypris rarus*) Compared to Individual Exposures, Aquatic Toxicology. (2025) 285, 10.1016/j.aquatox.2025.107416. 40412111

[12] Zulkipli S. Z. , Liew H. J. , Ando M. et al., A Review of Mercury Pathological Effects on Organs Specific of Fishes, Environmental Pollutants and Bioavailability. (2021) 33, no. 1, 76–87, 10.1080/26395940.2021.1920468.

[13] Trivedi S. P. , Singh S. , Trivedi A. , and Kumar M. , Mercuric Chloride‐Induced Oxidative Stress, Genotoxicity, Haematological Changes and Histopathological Alterations in Fish *Channa punctatus* (B Loch, 1793), Journal of Fish Biology. (2022) 100, no. 4, 868–883, 10.1111/jfb.15019. 35195905

[14] Liu S. , Huang J. , Zhang W. et al., Microplastics as a Vehicle of Heavy Metals in Aquatic Environments: A Review of Adsorption Factors, Mechanisms, and Biological Effects, Journal of Environmental Management. (2022) 302, 10.1016/j.jenvman.2021.113995. 34700080

[15] Menéndez-Pedriza A. and Jaumot J. , Interaction of Environmental Pollutants With Microplastics: A Critical Review of Sorption Factors, Bioaccumulation and Ecotoxicological Effects, Toxics. (2020) 8, no. 2, 10.3390/toxics8020040. PMC7355763 32498316

[16] Sunny A. R. , Sazzad S. A. , Islam M. A. et al., Microplastics in Aquatic Ecosystems: A Global Review of Distribution, Ecotoxicological Impacts, and Human Health Risks, Water. (2025) 17, no. 12, 10.3390/w17121741.

[17] Cheng C. , Tian W. , Wu Y. et al., Microplastics Have Additive Effects on Cadmium Accumulation and Toxicity in Rice Flower Carp (*Procypris merus*), Science of the Total Environment. (2024) 930, 10.1016/j.scitotenv.2024.172679. 38677436

[18] Subaramaniyam U. , Allimuthu R. S. , Vappu S. et al., Effects of Microplastics, Pesticides and Nano-Materials on Fish Health, Oxidative Stress and Antioxidant Defense Mechanism, Frontiers in Physiology. (2023) 14, 10.3389/fphys.2023.1217666. PMC10331820 37435307

[19] Fathy M. , Mohamed I. A. , Farghal A. I. A. , Temerak S. A. H. , and Sayed A. E.-D. H. , Hemotoxic Effects of Some Herbicides on Juvenile of Nile Tilapia *Oreochromis niloticus* , Environmental Science & Pollution Research. (2019) 26, no. 30, 30857–30865, 10.1007/s11356-019-06280-x. 31446602

[20] Mekkawy I. A. , Mahmoud U. M. , Hana M. N. , and Sayed A. E.-D. H. , Cytotoxic and Hemotoxic Effects of Silver Nanoparticles on the African Catfish, *Clarias gariepinus* (Burchell, 1822), Ecotoxicology and Environmental Safety. (2019) 171, 638–646, 10.1016/j.ecoenv.2019.01.011. 30658299

[21] Shahjahan M. , Rahman M. S. , Islam S. M. M. , Uddin M. H. , and Al-Emran M. , Increase in Water Temperature Increases Acute Toxicity of Sumithion Causing Nuclear and Cellular Abnormalities in Peripheral Erythrocytes of Zebrafish *Danio rerio* , Environmental Science & Pollution Research. (2019) 26, no. 36, 36903–36912, 10.1007/s11356-019-06886-1. 31745778

[22] Suchana S. A. , Ahmed M. S. , Islam S. M. M. et al., Chromium Exposure Causes Structural Aberrations of Erythrocytes, Gills, Liver, Kidney, and Genetic Damage in Striped Catfish *Pangasianodon hypophthalmus* , Biological Trace Element Research. (2021) 199, no. 10, 3869–3885, 10.1007/s12011-020-02490-4. 33206307

[23] Jaies I. , Shah F. A. , Qadiri S. S. N. et al., Immunological and Molecular Diagnostic Techniques in Fish Health: Present and Future Prospectus, Molecular Biology Reports. (2024) 51, no. 1, 10.1007/s11033-024-09344-5. 38642170

[24] Zhang F. , Li D. , Yang Y. et al., Combined Effects of Polystyrene Microplastics and Copper on Antioxidant Capacity, Immune Response and Intestinal Microbiota of Nile Tilapia (*Oreochromis niloticus*), Science of the Total Environment. (2022) 808, 10.1016/j.scitotenv.2021.152099. 34863761

[25] Getnet M. A. , Mekonnen M. Y. , Yimam H. M. , Berihun A. M. , and Malede B. A. , Histopathology Based Study of Nile Tilapia Fish (*Oreochromis niloticus*) as a Biomarker for Water Pollution Evaluation in the Southern Gulf of Lake Tana, Ethiopia, BMC Veterinary Research. (2024) 20, no. 1, 10.1186/s12917-024-04230-5. PMC11391847 39267064

[26] Booth A. M. and Sørensen L. , Microplastic Fate and Impacts in the Environment, Handb. Microplastics Environ, 2020, Springer International Publishing, Cham, 1–24, 10.1007/978-3-030-10618-8_29-1.

[27] Hasan J. , Siddik M. A. , Ghosh A. K. , Mesbah S. B. , Sadat M. A. , and Shahjahan M. , Increase in Temperature Increases Ingestion and Toxicity of Polyamide Microplastics in Nile Tilapia, Chemosphere. (2023) 327, 10.1016/j.chemosphere.2023.138502. 36965532

[28] Jasim M. A. , Sofian-Azirun M. , Yusoff I. , and Rahman M. M. , Bioaccumulation and Histopathological Changes Induced by Toxicity of Mercury (HgCl_2_) to Tilapia Fish *Oreochromis niloticus* , Sains Malaysiana. (2016) 45, 119–127.

[29] Xie M. , Lin L. , Xu P. et al., Effects of Microplastic Fibers on *Lates calcarifer* Juveniles: Accumulation, Oxidative Stress, Intestine Microbiome Dysbiosis and Histological Damage, Ecological Indicators. (2021) 133, 10.1016/j.ecolind.2021.108370.

[30] Nikki R. , Abdul Jaleel K. U. , Ragesh S. , Shini S. , Saha M. , and Dinesh Kumar P. K. , Abundance and Characteristics of Microplastics in Commercially Important Bottom Dwelling Finfishes and Shellfish of the Vembanad Lake, India, Marine Pollution Bulletin. (2021) 172, 10.1016/j.marpolbul.2021.112803. 34371342

[31] OECD , Test No. 215: Fish, Juvenile Growth Test, 2000, 10.1787/9789264070202-en.

[32] Shahjahan M. , Taslima K. , Rahman M. S. , Al-Emran M. , Alam S. I. , and Faggio C. , Effects of Heavy Metals on Fish Physiology—A Review, Chemosphere. (2022) 300, 10.1016/j.chemosphere.2022.134519. 35398071

[33] Mercy F. T. , Alam A. K. M. R. , and Akbor M. A. , Abundance and Characteristics of Microplastics in Major Urban Lakes of Dhaka, Bangladesh, Heliyon. (2023) 9, no. 4, 10.1016/j.heliyon.2023.e14587. PMC10073641 37035360

[34] Riya K. K. , Anisuzzaman M. , Samad Azad M. A. et al., Characteristics, Contamination Levels, and Ecosystem Risk Assessment of Microplastics in Surface Water of a Highly Urbanized River From a Developing Country, ACS Omega. (2024) 9, no. 52, 50922–50932, 10.1021/acsomega.4c01528. 39758629 PMC11696409

[35] Feldman A. T. and Wolfe D. , Tissue Processing and Hematoxylin and Eosin Staining, Methods in Molecular Biology. (2014) 31–43, 10.1007/978-1-4939-1050-2_3. 25015141

[36] Islam M. , Khanom H. , Islam N. et al., Probiotics and Spirulina platensis Improved Growth Performance of Nile Tilapia (*Oreochromis niloticus*) by Upgrading Intestinal Morphology and Activating GH/IGF Axis, Aquaculture Research. (2025) 2025, no. 1, 10.1155/are/1839162.

[37] Amin M. , Ahamed M. , Islam M. et al., Spirulina platensis Supplementation Remediates Microplastics-Induced Growth Inhibition and Stress in Nile Tilapia, *Oreochromis niloticus* , Journal of Hazardous Materials Advances. (2025) 18, 10.1016/j.hazadv.2025.100754.

[38] Batalhão I. G. , Lima D. , Russi A. P. M. et al., Effects of Methylphenidate on the Aggressive Behavior, Serotonin and Dopamine Levels, and Dopamine-Related Gene Transcription in Brain of Male Nile Tilapia (*Oreochromis niloticus*), Fish Physiology and Biochemistry. (2019) 45, no. 4, 1377–1391, 10.1007/s10695-019-00645-2. 31054043

[39] Rohani M. F. , Pesticides Toxicity in Fish: Histopathological and Hemato-Biochemical Aspects—A Review, Emerging Contaminants. (2023) 9, no. 3, 10.1016/j.emcon.2023.100234.

[40] Oladimeji T. E. , Oyedemi M. , Emetere M. E. , Agboola O. , Adeoye J. B. , and Odunlami O. A. , Review on the Impact of Heavy Metals From Industrial Wastewater Effluent and Removal Technologies, Heliyon. (2024) 10, no. 23, 10.1016/j.heliyon.2024.e40370. PMC11625160 39654720

[41] Ding J. , Li J. , Sun C. et al., An Examination of the Occurrence and Potential Risks of Microplastics Across Various Shellfish, Science of the Total Environment. (2020) 739, 10.1016/j.scitotenv.2020.139887. 32758939

[42] Tumwesigye E. , Felicitas Nnadozie C. , C Akamagwuna F. , Siwe Noundou X. , William Nyakairu G. , and Odume O. N. , Microplastics as Vectors of Chemical Contaminants and Biological Agents in Freshwater Ecosystems: Current Knowledge Status and Future Perspectives, Environment and Pollution. (2023) 330, 10.1016/j.envpol.2023.121829. 37196837

[43] Li Z. , Zhao M. , Feng Z. et al., Combined Toxicity of Polyvinyl Chloride Microplastics and Copper to Marine Jacopever (*Sebastes schlegelii*), Marine Environment Research. (2024) 199, 10.1016/j.marenvres.2024.106598. 38865873

[44] Chen Q. , Zhao H. , Liu Y. , Jin L. , and Peng R. , Factors Affecting the Adsorption of Heavy Metals by Microplastics and Their Toxic Effects on Fish, Toxics. (2023) 11, no. 6, 10.3390/toxics11060490. PMC10305669 37368590

[45] Liu S. , Shi J. , Wang J. et al., Interactions Between Microplastics and Heavy Metals in Aquatic Environments: A Review, Frontiers in Microbiology. (2021) 12, 10.3389/fmicb.2021.652520. PMC8100347 33967988

[46] Li B. , Liang W. , Liu Q.-X. et al., Fish Ingest Microplastics Unintentionally, Environmental Science and Technology. (2021) 55, no. 15, 10471–10479, 10.1021/acs.est.1c01753. 34297559

[47] Capparelli M. V. , Dzul-Caamal R. , Rodríguez-Cab E. M. et al., Synergistic Effects of Microplastic and Lead Trigger Physiological and Biochemical Impairment in a Mangrove Crab, Comparative Biochemistry and Physiology, Part C: Toxicology & Pharmacology. (2024) 276, 10.1016/j.cbpc.2023.109809. 38056684

[48] Islam N. , Shahriar S. I. M. , Noor S. A. , Paray B. A. , Zahangir M. M. , and Shahjahan M. , Alteration of Growth, Hematology, Histopathology of Tissues and Immune-Antioxidant Genes Expression in Nile Tilapia Following Co-Exposure of Hexavalent Chromium and Polyamide Microplastics, Ecotoxicology. (2025) 34, no. 7, 1300–1311, 10.1007/s10646-025-02917-5. 40571808

[49] Zitouni N. , Bousserrhine N. , Missawi O. et al., Uptake, Tissue Distribution and Toxicological Effects of Environmental Microplastics in Early Juvenile Fish *Dicentrarchus labrax* , Journal of Hazardous Materials. (2021) 403, 10.1016/j.jhazmat.2020.124055. 33265060

[50] Bai Z. , He Y. , Hu G. , Cheng L. , and Wang M. , Microplastics at an Environmentally Relevant Dose Enhance Mercury Toxicity in a Marine Copepod Under Multigenerational Exposure: Multi-Omics Perspective, Journal of Hazardous Materials. (2024) 478, 10.1016/j.jhazmat.2024.135529. 39154477

[51] Lei Y. , Li X. , and Mao X. , Microplastics Aggravate the Adverse Effects of Methylmercury Than Inorganic Mercury on Zebrafish (*Danio rerio*), Environment and Pollution. (2025) 367, 10.1016/j.envpol.2024.125559. 39710179

[52] Abbaszadeh M. , Sayadi M. H. , and Kharkan J. , Impact of Polyvinyl Chloride Microplastic and Paraquat Herbicide on the Blood Cells, Biochemical Parameters, Liver Enzymes and Morphological Changes of Aqueduct Fish, Chemosphere. (2024) 362, 10.1016/j.chemosphere.2024.142643. 38897326

[53] Shahjahan M. , Islam M. J. , Hossain M. T. , Mishu M. A. , Hasan J. , and Brown C. , Blood Biomarkers as Diagnostic Tools: An Overview of Climate-Driven Stress Responses in Fish, Science of the Total Environment. (2022) 843, 10.1016/j.scitotenv.2022.156910. 35753474

[54] Soliman H. A. M. , Salaah S. M. , Hamed M. , and Sayed A. E.-D. H. , Toxicity of Co-Exposure of Microplastics and Lead in African Catfish (*Clarias gariepinus*), Frontiers in Veterinary Science. (2023) 10, 10.3389/fvets.2023.1279382. PMC10588188 37869502

[55] Zhang Y. , Wang C. , Jia R. et al., Transfer From Ciliate to Zebrafish: Unveiling Mechanisms and Combined Effects of Microplastics and Heavy Metals, Journal of Hazardous Materials. (2024) 479, 10.1016/j.jhazmat.2024.135645. 39191009

[56] Haque M. A. , Bin Anwar M. , Hossain A. A. , Ahmed S. , Zahangir M. M. , and Shahjahan M. , Co-exposure Toxicity of Microplastic and Sumithion in Nile Tilapia – Changes in Growth, Hematology, Histopathology of Internal Tissues and Immune-Antioxidant Genes Expression, Journal of Hazardous Materials Advances. (2025) 19, 10.1016/j.hazadv.2025.100840.

[57] Xiao K. , Wang X. , Liu W.-B. et al., Corticosterone Can Be an Essential Stress Index in Channel Catfish (*Ictalurus punctatus*), Frontiers in Marine Science. (2021) 8, 10.3389/fmars.2021.692726.

[58] Ahmed I. , Zakiya A. , and Fazio F. , Effects of Aquatic Heavy Metal Intoxication on the Level of Hematocrit and Hemoglobin in Fishes: A Review, Frontiers of Environmental Science. (2022) 10, 10.3389/fenvs.2022.919204.

[59] Khatun M. M. , Mostakim G. M. , Moniruzzaman M. , Rahman U. O. , and Islam M. S. , Distortion of Micronuclei and Other Peripheral Erythrocytes Caused by Fenitrothion and Their Recovery Assemblage in Zebrafish, Toxicology Reports. (2021) 8, 415–421, 10.1016/j.toxrep.2021.02.019. 33680864 PMC7930503

[60] Hovhannisyan G. , Harutyunyan T. , and Aroutiounian R. , Micronuclei and what They Can Tell Us in Cytogenetic Diagnostics, Current Genetic Medicine Reports. (2018) 6, no. 4, 144–154, 10.1007/s40142-018-0149-6.

[61] Trivedi S. P. , Ratn A. , Awasthi Y. , Gupta N. , Kumar M. , and Trivedi A. , Micronuclei and Other Nuclear Abnormalities in Phorate Exposed Fish, *Channa punctatus* , Journal of Environmental Biology. (2021) 42, no. 5, 1221–1231, 10.22438/jeb/42/5/MRN-1748.

[62] Sharmin S. , Islam M. T. , Sadat M. A. , Jannat R. , Alam M. R. , and Shahjahan M. , Sumithion Induced Structural Erythrocyte Alteration and Damage to the Liver and Kidney of Nile Tilapia, Environmental Science & Pollution Research. (2021) 28, no. 27, 36695–36706, 10.1007/s11356-021-13263-4. 33694120

[63] Zuo Z. , Wang Q. , Zhang C. , and Zou J. , Single and Combined Effects of Microplastics and Cadmium on Juvenile Grass Carp (*Ctenopharyngodon idellus*), Comparative Biochemistry and Physiology, Part C: Toxicology & Pharmacology. (2022) 261, 10.1016/j.cbpc.2022.109424. 35918021

[64] Marshoudi M. A. , Al Reasi H. A. , Al-Habsi A. , and Barry M. J. , Additive Effects of Microplastics on Accumulation and Toxicity of Cadmium in Male Zebrafish, Chemosphere. (2023) 334, 10.1016/j.chemosphere.2023.138969. 37244557

[65] Kim H. J. , Shin S. R. , Park J. J. , and Lee J. S. , Feeding, Excretion, Survival, and Histological Alterations in Zebrafish *Danio rerio* from Single and Combined Exposure to Microplastics and Copper, Environ. Biol. Res.(2024) 42, 1–14, 10.11626/KJEB.2024.42.1.001.

[66] Wang S. , Xie S. , Wang Z. et al., Single and Combined Effects of Microplastics and Cadmium on the Cadmium Accumulation and Biochemical and Immunity of *Channa argus* , Biological Trace Element Research. (2022) 200, no. 7, 3377–3387, 10.1007/s12011-021-02917-6. 34564831

[67] Barbieri E. , Campos-Garcia J. , Martinez D. S. T. , da Silva J. R. M. C. , Alves O. L. , and Rezende K. F. O. , Histopathological Effects on Gills of Nile Tilapia (*Oreochromis niloticus*, Linnaeus, 1758) Exposed to Pb and Carbon Nanotubes, Microscopy and Microanalysis. (2016) 22, no. 6, 1162–1169, 10.1017/S1431927616012009. 27998365

[68] Singh G. and Sharma S. , Heavy Metal Contamination in Fish: Sources, Mechanisms and Consequences, Aquatic Sciences. (2024) 86, no. 4, 10.1007/s00027-024-01121-7.

[69] Dane H. and Şi̇şman T. , A Morpho-Histopathological Study in the Digestive Tract of Three Fish Species Influenced with Heavy Metal Pollution, Chemosphere. (2020) 242, 10.1016/j.chemosphere.2019.125212. 31677508

[70] Karbalaei S. , Hanachi P. , Rafiee G. , Seifori P. , and Walker T. R. , Toxicity of Polystyrene Microplastics on Juvenile *Oncorhynchus mykiss* (Rainbow Trout) After Individual and Combined Exposure with Chlorpyrifos, Journal of Hazardous Materials. (2021) 403, 10.1016/j.jhazmat.2020.123980. 33265019

[71] Li X. , Jing K. , Song P. , and Yu J. , Aged Polystyrene Microplastics Exacerbate Cadmium-Induced Hepatotoxicity in Zebrafish Through Gut-Liver Axis Metabolic Dysregulation, Environmental Chemistry and Ecotoxicology. (2025) 7, 859–871, 10.1016/j.enceco.2025.05.001.

[72] Chen Q.-L. , Sun Y.-L. , Liu Z.-H. , and Li Y.-W. , Sex-Dependent Effects of Subacute Mercuric Chloride Exposure on Histology, Antioxidant Status and Immune-Related Gene Expression in the Liver of Adult Zebrafish (*Danio rerio*), Chemosphere. (2017) 188, 1–9, 10.1016/j.chemosphere.2017.08.148. 28865787

[73] Brancatelli G. , Furlan A. , Calandra A. , and Dioguardi Burgio M. , Hepatic Sinusoidal Dilatation, Abdominal Radiology. (2018) 43, no. 8, 2011–2022, 10.1007/s00261-018-1465-8. 29392360

[74] Chen X. , Wang J. , Xie Y. et al., Physiological Response and Oxidative Stress of Grass Carp (*Ctenopharyngodon idellus*) Under Single and Combined Toxicity of Polystyrene Microplastics and Cadmium, Ecotoxicology and Environmental Safety. (2022) 245, 10.1016/j.ecoenv.2022.114080. 36152428

[75] Radi Z. A. , Kidney Pathophysiology, Toxicology, and Drug-Induced Injury in Drug Development, International Journal of Toxicology. (2019) 38, no. 3, 215–227, 10.1177/1091581819831701. 30845865

[76] Bakhasha J. , Saxena V. , Arya N. , Kumar P. , and Trivedi A. , Metalloplastic Interaction Triggers Renal Oxeiptosis: Novel Insights Into KEAP1/PGAM5/AIFM1 Pathway in Snakeheaded Fish *Channa punctatus* , Environmental Toxicology and Pharmacology. (2025) 118, 10.1016/j.etap.2025.104760. 40681132

[77] Mozafarjalali M. , Hamidian A. H. , and Sayadi M. H. , Microplastics as Carriers of Iron and Copper Nanoparticles in Aqueous Solution, Chemosphere. (2023) 324, 10.1016/j.chemosphere.2023.138332. 36893866

[78] Zhang X. , Chen X. , Gao L. et al., Transgenerational Effects of Microplastics on Nrf2 Signaling, GH/IGF, and HPI Axis in Marine Medaka *Oryzias melastigma* Under Different Salinities, Science of the Total Environment. (2024) 906, 10.1016/j.scitotenv.2023.167170. 37730060

[79] Zhong H. , Zhou Y. , Liu S. et al., Elevated Expressions of GH/IGF Axis Genes in Triploid Crucian Carp, General and Comparative Endocrinology. (2012) 178, no. 2, 291–300, 10.1016/j.ygcen.2012.06.006. 22713693

[80] Luckenbach J. A. , Dickey J. T. , and Swanson P. , Regulation of Pituitary GnRH Receptor and Gonadotropin Subunits by IGF1 and GnRH in Prepubertal Male Coho Salmon, General and Comparative Endocrinology. (2010) 167, no. 3, 387–396, 10.1016/j.ygcen.2009.09.010. 19800342

[81] de la Serrana D. G. and Macqueen D. J. , Insulin-Like Growth Factor-Binding Proteins of Teleost Fishes, Frontiers in Endocrinology. (2018) 9, 10.3389/fendo.2018.00080. PMC5857546 29593649

[82] Canosa L. F. and Bertucci J. I. , The Effect of Environmental Stressors on Growth in Fish and Its Endocrine Control, Frontiers in Endocrinology. (2023) 14, 10.3389/fendo.2023.1109461. PMC10098185 37065755

[83] Chen X. , Peng L. B. , Wang D. , Zhu Q. L. , and Zheng J. L. , Combined Effects of Polystyrene Microplastics and Cadmium on Oxidative Stress, Apoptosis, and GH/IGF Axis in Zebrafish Early Life Stages, Science of the Total Environment. (2022) 813, 10.1016/j.scitotenv.2021.152514. 34968615

[84] Trivedi A. , Bakhasha J. , Yadav K. K. , Saxena V. , Neeti , and Yadav S. , Zinc Induced Oxidative Stress and Hepatoarchitectural Changes in Fresh Water Fish *Channa punctatus* , Biotech Today. (2023) 13, no. 1, 23–30, 10.5958/2322-0996.2023.00004.2.

[85] Ahmadifar E. , Kalhor N. , Dawood M. A. O. et al., Effects of Polystyrene Microparticles on Inflammation, Antioxidant Enzyme Activities, and Related Gene Expression in Nile Tilapia (*Oreochromis niloticus*), Environmental Science & Pollution Research. (2021) 28, no. 12, 14909–14916, 10.1007/s11356-020-11731-x. 33222065

[86] Wang S. , Xie S. , Zhang C. et al., Interactions Effects of Nano-Microplastics and Heavy Metals in Hybrid Snakehead (*Channa maculata* ♀ × *Channa argus* ♂), Fish & Shellfish Immunology. (2022) 124, 74–81, 10.1016/j.fsi.2022.03.045. 35378307

[87] Chen M. , Bao X. , Yue Y. et al., Combined Effects of Cadmium and Nanoplastics on Oxidative Stress, Histopathology, and Intestinal Microbiota in Largemouth Bass (*Micropterus salmoides*), Aquaculture. (2023) 569, 10.1016/j.aquaculture.2023.739363.

[88] Lee M. T. , Lin W. C. , Yu B. , and Lee T. T. , Antioxidant Capacity of Phytochemicals and Their Potential Effects on Oxidative Status in Animals—A Review, Asian-Australasian Journal of Animal Sciences. (2016) 30, no. 3, 299–308, 10.5713/ajas.16.0438. 27660026 PMC5337908

[89] Koner D. , Banerjee B. , Kumari A. , Lanong A. S. , Snaitang R. , and Saha N. , Molecular Characterization of Superoxide Dismutase and Catalase Genes, and the Induction of Antioxidant Genes Under the Zinc Oxide nanoparticle-induced Oxidative Stress in Air-Breathing Magur Catfish (*Clarias magur*), Fish Physiology and Biochemistry. (2021) 47, no. 6, 1909–1932, 10.1007/s10695-021-01019-3. 34609607

[90] Bartee E. , Mohamed M. R. , and McFadden G. , Tumor Necrosis Factor and Interferon: Cytokines in Harmony, Current Opinion in Microbiology. (2008) 11, no. 4, 378–383, 10.1016/j.mib.2008.05.015. 18595771 PMC7108444

[91] Whyte S. K. , The Innate Immune Response of Finfish—A Review of Current Knowledge, Fish & Shellfish Immunology. (2007) 23, no. 6, 1127–1151, 10.1016/j.fsi.2007.06.005. 17980622

[92] Giambò F. , Leone G. M. , Gattuso G. et al., Genetic and Epigenetic Alterations Induced by Pesticide Exposure: Integrated Analysis of Gene Expression, microRNA Expression, and DNA Methylation Datasets, International Journal of Environmental Research and Public Health. (2021) 18, no. 16, 10.3390/ijerph18168697. PMC8394939 34444445

[93] López-García L. and Castro-Manrreza M. E. , TNF-α and IFN-γ Participate in Improving the Immunoregulatory Capacity of Mesenchymal Stem/Stromal Cells: Importance of Cell–Cell Contact and Extracellular Vesicles, International Journal of Molecular Sciences. (2021) 22, no. 17, 10.3390/ijms22179531. PMC8431422 34502453

[94] Li W. , Liu Q. , Shi J. , Xu X. , and Xu J. , The Role of TNF-α in the Fate Regulation and Functional Reprogramming of Mesenchymal Stem Cells in an Inflammatory Microenvironment, Frontiers in Immunology. (2023) 14, 10.3389/fimmu.2023.1074863. PMC9940754 36814921

[95] Cao J. , Xu R. , Wang F. et al., Polyethylene Microplastics Trigger Cell Apoptosis and Inflammation via Inducing Oxidative Stress and Activation of the NLRP3 Inflammasome in Carp Gills, Fish & Shellfish Immunology. (2023) 132, 10.1016/j.fsi.2022.108470. 36470402

